# Genome-Wide Expression Profiling of Five Mouse Models Identifies Similarities and Differences with Human Psoriasis

**DOI:** 10.1371/journal.pone.0018266

**Published:** 2011-04-04

**Authors:** William R. Swindell, Andrew Johnston, Steve Carbajal, Gangwen Han, Christian Wohn, Jun Lu, Xianying Xing, Rajan P. Nair, John J. Voorhees, James T. Elder, Xiao-Jing Wang, Shigetoshi Sano, Errol P. Prens, John DiGiovanni, Mark R. Pittelkow, Nicole L. Ward, Johann E. Gudjonsson

**Affiliations:** 1 Department of Genetics, Harvard Medical School, Boston, Massachusetts, United States of America; 2 Department of Dermatology, University of Michigan Medical School, Ann Arbor, Michigan, United States of America; 3 Division of Pharmacology & Toxicology, College of Pharmacy, The University of Texas at Austin, Austin, Texas, United States of America; 4 Departments of Pathology, Otolaryngology and Dermatology, University of Colorado, Denver, Colorado, United States of America; 5 Departments of Immunology, Erasmus MC, University Medical Center, Rotterdam, The Netherlands; 6 Department of Dermatology, Mayo Clinic, Rochester, Minnesota, United States of America; 7 Ann Arbor Veterans Affairs Hospital, Ann Arbor, Michigan, United States of America; 8 Department of Dermatology, Kochi Medical School, Kochi University, Okocho, Nankoku, Japan; 9 Departments of Dermatology and Rheumatology, Erasmus MC, University Medical Center, Rotterdam, The Netherlands; 10 Department of Nutritional Sciences, Dell Pediatric Research Institute, The University of Texas at Austin, Austin, Texas, United States of America; 11 Division of Pharmacology & Toxicology, Dell Pediatric Research Institute, The University of Texas at Austin, Austin, Texas, United States of America; 12 Department of Dermatology and the Murdough Family Center for Psoriasis, Case Western Reserve University and University Hospitals, Case Medical Center, Cleveland, Ohio, United States of America; Charité-University Medicine Berlin, Germany

## Abstract

Development of a suitable mouse model would facilitate the investigation of pathomechanisms underlying human psoriasis and would also assist in development of therapeutic treatments.

However, while many psoriasis mouse models have been proposed, no single model recapitulates all features of the human disease, and standardized validation criteria for psoriasis mouse models have not been widely applied. In this study, whole-genome transcriptional profiling is used to compare gene expression patterns manifested by human psoriatic skin lesions with those that occur in five psoriasis mouse models (K5-Tie2, imiquimod, K14-AREG, K5-Stat3C and K5-TGFbeta1). While the cutaneous gene expression profiles associated with each mouse phenotype exhibited statistically significant similarity to the expression profile of psoriasis in humans, each model displayed distinctive sets of similarities and differences in comparison to human psoriasis. For all five models, correspondence to the human disease was strong with respect to genes involved in epidermal development and keratinization. Immune and inflammation-associated gene expression, in contrast, was more variable between models as compared to the human disease. These findings support the value of all five models as research tools, each with identifiable areas of convergence to and divergence from the human disease. Additionally, the approach used in this paper provides an objective and quantitative method for evaluation of proposed mouse models of psoriasis, which can be strategically applied in future studies to score strengths of mouse phenotypes relative to specific aspects of human psoriasis.

## Introduction

Psoriasis is a chronic inflammatory disease that leads to widespread development of erythematous plaques with adherent silvery scales. The disease is believed to be primarily mediated by T-cells, which release cytokines that stimulate keratinocyte (KC) hyperproliferation and altered differentiation [1], [2]. Development of a single suitable animal model would greatly facilitate research on the mechanism(s) of action that drive inflammatory and autoimmune processes associated with psoriasis [3]–[5]. Such an animal model, for example, would permit experiments using genetically uniform subjects within a controlled environment, as well as high-throughput screening of potential therapeutic agents. Most clinical features of psoriasis, however, arise spontaneously only in humans and closely related primate species [6], [7]. The laboratory mouse offers the most flexible experimental system for development of new psoriasiform phenotypes and previous studies have described transgenic or deletion mutants with skin conditions that resemble human clinical psoriasis [3]–[5]. Given the many disparities between human and mouse skin, it cannot be expected that psoriasiform phenotypes in mice will mirror the human disease in every respect. For instance, relative to human skin, mouse skin has a denser distribution of hair follicles, thinner epidermis, and an underlying cutaneous muscle layer that is generally absent in humans [4], [8], [9]. Additionally, mice possess subsets of inflammatory cells that are absent in humans [10], . Despite these challenges, psoriasis mouse models have already provided mechanistic insights into inflammatory skin diseases [12]–[16].

An ideal model would realistically recapitulate a marked epidermal hyperproliferation, thickening and altered differentiation of the epidermis, an inflammatory infiltrate that includes T-cells, altered vascularity, and responsiveness to current anti-psoriatic therapies [3]–[5]. Mouse phenotypes that satisfy a majority of these characteristics include; overexpression of the endothelial-specific receptor tyrosine kinase in basal KCs (K5-Tie2) [12], topical application of the toll-like receptor (TLR) agonist imiquimod (IMQ) [13], overexpression of human amphiregulin in the basal epidermal layer (K14-AREG) [14], basal KC-specific overexpression of a constitutively active mutant of signal transducer and activator of transcription 3 (K5-Stat3C) [15], and overexpression of the latent form of transforming growth factor beta 1 in basal KCs (K5-TGFβ1) [16]. The phenotypes of these models recapitulate key features of human psoriasis, but differ with respect to the initiating (biochemical) events. The K14-AREG and K5-Stat3C models involve epidermal overexpression of a growth factor and signaling component, respectively, which directly perturbs KC homeostasis, leading to elevated cytokine or chemokine production and secondary inflammatory responses [14], [15]. The K5-Tie2 and K5-TGFβ1phenotypes may also arise, in part, from a direct perturbation of KC homeostasis; but in these models, a role has also been postulated for growth factor release and certain concurrent processes (e.g., angiogenesis, oxidative stress accumulation, and/or basement membrane degradation), which may also contribute to KC proliferation and initiation of inflammatory cascades [12], [16]. By comparison, IMQ-treated mice develop a psoriasiform phenotype that may differ in fundamental ways, since the phenotype arises from direct (over)stimulation of the immune system. IMQ is a TLR7 agonist that drives skin inflammation and immune cell infiltration, followed by KC proliferation and enhanced dermal vascularity [13]. In mice, these effects of IMQ produce red, scaly skin similar to human psoriasis, and indeed, clinical observations have indicated that IMQ can exacerbate psoriasis in patients [17]–[20].

The five psoriasiform phenotypes discussed above represent potentially useful tools for studying human psoriasis. However, further characterization of each model at the biochemical level is needed for refinement and development of mouse models with stronger similarity to clinical disease [3]–[5]. Along these lines, it has been challenging to align separate analyses performed by different laboratories, and no side-by-side evaluation has previously assessed each model using a common methodological strategy. Some investigators have closely followed a set of proposed guidelines for validation of psoriasis mouse models, which are based on phenotypic measures (e.g., acanthosis, vascularity, absence of papillomatosis) and experimental demonstration (e.g., T-cell dependence and drug response) [13]. This strategy has been valuable, when applied, but is at best semi-quantitative and does not clearly differentiate mouse phenotypes that satisfy most or all of the proposed standards (e.g., K5-Tie2 and K5-Stat3C). For this reason, phenotypic and experimental approaches should be complemented, but not replaced, by additional strategies. Genome-wide transcriptional profiling is an example of one such complementary approach. Microarray analyses have identified dramatic differences between psoriatic and normal skin from patients, which involve altered expression of hundreds of genes [21]–[24], and several recent large-scale genome-wide association studies have confidently identified 25 psoriasis susceptibility loci [25]–[30]. These findings underscore the polygenic basis of psoriasis and suggest that pathogenesis involves numerous biochemical pathways. An expression profiling approach can provide a “big picture” characterization of this process and support development of quantifiable metrics for evaluating similarity between human psoriasis and mouse phenotypes, allowing investigators to determine whether correspondence between human and mouse model psoriatic phenotypes is larger than expected on the basis of chance alone, and to decompose the global transcriptional correspondence into finer parts and evaluate correspondence with respect to specific pathways and pre-defined gene categories. Furthermore, microarray-based evaluation facilitates objective evaluation of mouse phenotypes, without “dependent variable selection bias” or over-emphasis of characteristics that best correspond between mouse phenotypes and human psoriasis.

In this study, global transcriptional profiling was utilized to evaluate the similarity between human psoriasis and the psoriasis-like phenotypes that develop in five mouse models (K5-Tie2, IMQ, K14-AREG, K5-Stat3C, K5-TGFβ1). A broad comparison is made between each mouse phenotype and clinical psoriasis on the basis of global gene expression patterns, along with more fine-grained comparisons that are specific to key psoriasis-associated biological processes and biochemical pathways. Additionally, for each mouse phenotype, we gauge the intensity of inflammation and composition of inflammatory infiltrate by analysis of leukocyte infiltration signatures embedded within genome-wide transcriptional response patterns [31]. These analyses provide the first transcriptomics-based assessment of correspondence between human psoriasis and multiple mouse phenotypes, and we suggest that similar analytic strategies can be adopted in future work to evaluate existing and new mouse models of psoriasis and other skin diseases.

## Results

### Gene expression patterns in psoriasis mouse models have broad and statistically significant resemblance to those of clinical psoriasis

**Figure 1:**
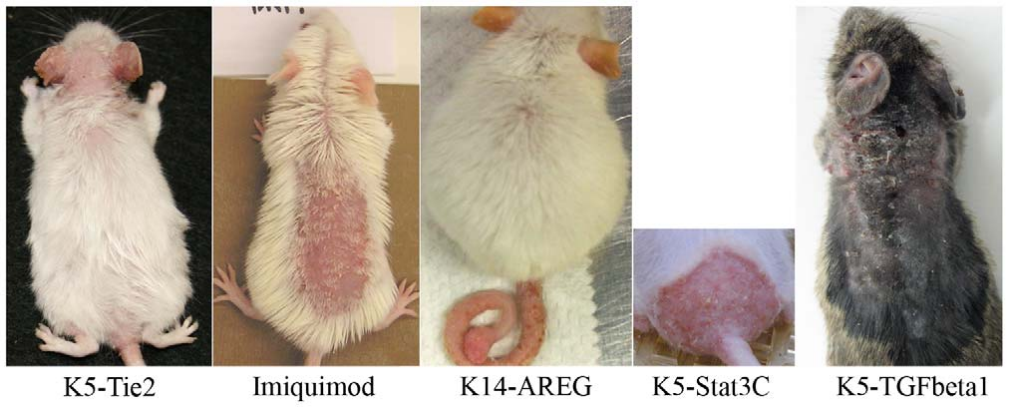
Psoriasiform phenotypes in the laboratory mouse. The figure shows representative images of the five psoriasis-like phenotypes evaluated in this study (i.e., K5-Tie2, IMQ, K14-AREG, K5-Stat3C and K5-TGFβ1). Each mouse model exhibits red, scaly skin with macroscopic features suggestive of and consistent with clinical psoriasis in humans.

**Figure 2:**
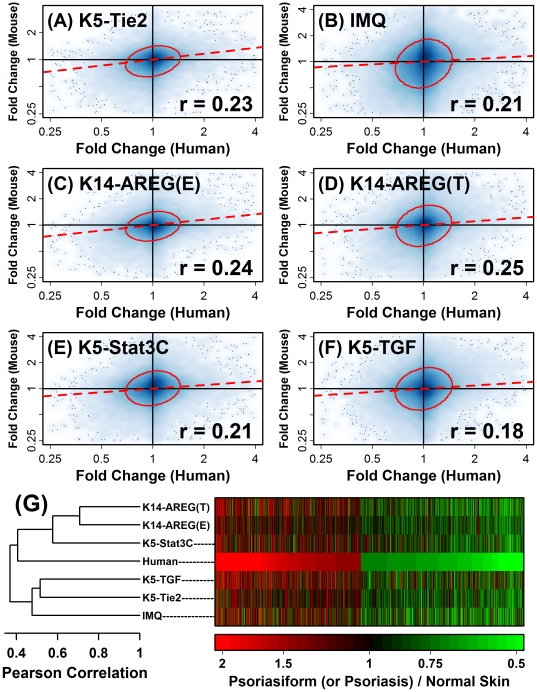
Global correspondence of gene expression between human psoriasis and mouse psoriasiform phenotypes. The global correlation was evaluated between gene expression patterns in human psoriasis and those in psoriasiform plaques obtained from (A) K5-Tie2 transgenic mice (back skin), (B) IMQ-treated mice (back skin), (C) K14-AREG mice (ear skin) (D) K14-AREG mice (tail skin), (E) K5-Stat3C mice (back skin) and (F) K5-TGFβ1 mice (back skin). Scatterplots shown in (A) - (F) are each based upon 33322 matching transcripts associated with orthologous human and mouse genes. For each transcript, the difference was calculated between its average expression across psoriatic skin samples from *n*  =  58 patients and its average expression across normal skin samples from a group of *n*  =  64 healthy subjects. The horizontal axis corresponds to the ratio of gene expression in psoriatic skin relative to skin from control subjects (log_2_ scale), with values larger than one indicating increased expression in psoriatic skin. The vertical axis corresponds to the ratio of gene expression in mouse psoriasiform skin relative to skin samples from control mice (log_2_ scale). The intensity of the blue shading represents the empirical density of 33322 points within the bivariate space, and the dotted red line was generated by least-squares regression. The red circle shown in each figure outlines the set of transcripts (75% of all transcripts) that are closest to the bivariate centroid (based upon Mahalanobis distance). The Pearson correlation coefficient (*r*) calculated from each scatterplot is shown in the lower-right corner of (A) - (F). In panel (G), the human-mouse correspondence was evaluated with respect to a subset of 2617 transcripts elevated in human psoriasis and 3540 transcripts decreased in human psoriasis (FDR-adjusted P<0.05, log_2_-transformed fold-change estimate greater than 0.50 in absolute value). Colors denote the fold-change estimate associated with human or mouse transcripts (see scale), with red regions indicating elevated expression in psoriatic skin or mouse phenotypes (i.e., fold-change greater than one), and green indicating decreased expression in psoriatic skin or mouse phenotypes (i.e., fold-change less than one). The expression profiles shown in the heatmap have been clustered according to the Pearson correlation coefficient (see dendrogram on left).

Whole-genome microarray analysis was used to identify transcripts altered in human psoriasis and each of five mouse psoriasiform phenotypes (back skin of K5-Tie2 transgenic mice, back skin of IMQ-treated mice, both ear and tail skin of K14-AREG transgenic mice, back skin of K5-Stat3C mice, and back skin of K5-TGFβ1 mice; see representative images of each phenotype in Figure 1). Expression patterns associated with human psoriasis were evaluated by comparing psoriatic skin from patients (*n* = 58) with normal skin from control subjects with no history of psoriasis (*n* = 64). Expression patterns associated with mouse phenotypes were evaluated by comparing lesional skin from transgenic or IMQ-treated mice (*n* = 2–3) with normal skin obtained from control mice (*n* = 2–3). Of 54,675 transcripts represented on the Affymetrix Human Genome U133 Plus 2.0 Array, approximately 61% (33,322) were matched with at least one transcript derived from an orthologous gene represented on the Affymetrix Mouse Genome 430 2.0 Array. With respect to these human-mouse transcript pairs, fold-change estimates associated with human psoriasis (psoriasis/control) were positively correlated with corresponding estimates in mouse phenotypes (psoriasiform/control) (Figures 2A–2F; 0.18≤ *r* ≤0.25). A cluster analysis grouped the K5-Tie2, IMQ-treated and K5-TGFβ1 mice apart from the K14-AREG and K5-Stat3C models, with the K14-AREG and K5-Stat3C phenotypes exhibiting slightly greater similarity to the expression pattern of human psoriasis (Figure 2G).

**Figure 3:**
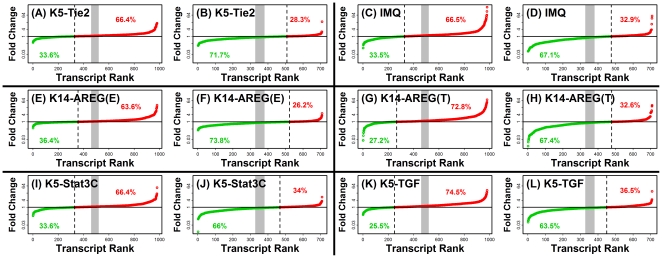
Statistically significant correspondence between human psoriasis and mouse psoriasiform phenotypes: Proportion of psoriasiform-increased to psoriasiform-decreased transcripts. Genes significantly increased or decreased in human psoriasis were identified and the expression of orthologous genes was studied in the K5-Tie2 phenotype (A and B), the IMQ phenotype (C and D), the K14-AREG phenotype on ear skin (E and F), the K14-AREG phenotype on tail skin (G and H), the K5-Stat3C phenotype (I and J), and the K5-TGFβ1 phenotype (K and L). We identified 793 transcripts with significantly elevated expression in human psoriasis (FDR-adjusted P<0.05 and log_2_-transformed fold-change estimate greater than 1.00), and these transcripts were associated with 981 transcripts derived from orthologous mouse genes. For these 981 mouse transcripts, the fold-change ratio was calculated between psoriasiform and normal mouse skin, and in figures A, C, E, G, I and K fold-change estimates have been ranked from smallest (left) to largest (right). Likewise, we identified 533 transcripts with significantly decreased expression in human psoriasis (FDR-adjusted P<0.05 and log_2_-transformed fold-change estimate less than 1.00 in absolute value), and these transcripts were associated with 709 transcripts derived from orthologous mouse genes. For these 709 transcripts, the fold-change ratio was calculated between psoriasiform skin and normal skin from control mice, and in figures B, D, F, H, J and L fold-change estimates have been ranked from smallest (left) to largest (right). In each figure, red symbols denote transcripts increased in psoriasiform mouse skin and green symbols denote transcripts decreased in psoriasiform skin. The dotted vertical line is equal to the number of psoriasiform-decreased transcripts, and the grey region corresponds to the random expectation, representing the 95% confidence limits associated with the null (hypergeometric) distribution. In figures A, C, E, G, I and K, there is a significant overabundance of psoriasiform-*increased* transcripts, because the dotted vertical line is left of the gray region. In figures B, D, F, H, J and L, there is a significant overabundance of psoriasiform-*decreased* transcripts, because the dotted vertical line is right of the gray region. Green and red percentage values shown in each figure indicate the percentage of psoriasiform-decreased and psoriasiform-increased transcripts, respectively.

**Figure 4:**
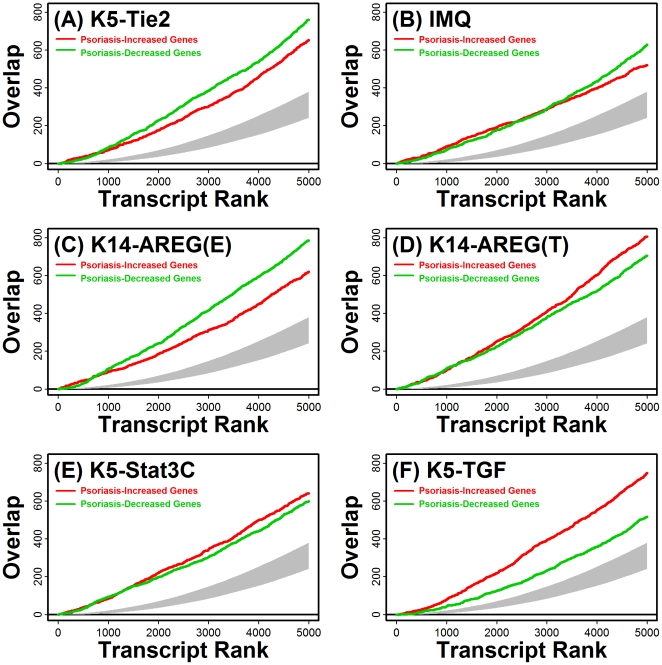
Statistically significant correspondence between human psoriasis and mouse psoriasiform phenotypes: Analysis of ranked gene lists. The 5000 transcripts with expression most strongly elevated in human psoriasis were identified, along with the 5000 transcripts with expression most strongly decreased in human psoriasis. These transcripts were ranked according to the estimated fold-change expression ratio (psoriasis/control), with lower ranks assigned to transcripts most strongly increased or decreased in human psoriasis. For any rank *N*, where *N* = 1, …, 5000, we isolated the top *N* human transcripts and identified orthologous mouse transcripts, and then determined whether these mouse transcripts overlapped significantly with the top *N* mouse transcripts increased or decreased in the (A) K5-Tie2 phenotype, (B) IMQ phenotype, (C) K14-AREG phenotype on ear skin, (D) K14-AREG phenotype on tail skin, (E) K5-Stat3C phenotype and (F) K5-TGFβ1 phenotype. In each figure, the red line corresponds to the overlap, at a given rank *N*, between the top *N* psoriasiform-increased mouse transcripts and the set of mouse transcripts orthologous to the top *N* psoriasis-increased human transcripts. Similarly, the green line corresponds to the overlap, at a given rank *N*, between the top *N* psoriasiform-decreased mouse transcripts and the set of mouse transcripts orthologous to the top *N* psoriasis-decreased human transcripts. The grey region outlines the level of overlap expected by chance for any given rank *N* (i.e., the 95% confidence region of the null hypergeometric distribution). A significant level of overlap is indicated for each psoriasiform phenotype because red and green lines lie above the grey region that spans the null expectation.

The global similarity between expression patterns in human psoriasis and mouse phenotypes was statistically significant for each mouse model evaluated. This was demonstrated by five different analytical approaches (Figures 3, 4, S1, S2 and S3). First, we asked whether mouse transcripts orthologous to genes with increased expression in human psoriasis were disproportionately elevated in psoriasiform phenotypes, and conversely, whether mouse orthologues of psoriasis-decreased genes were disproportionately decreased in psoriasiform phenotypes. This expectation was validated in both cases and with respect to each mouse phenotype considered (Figure 3; P<0.001 for each mouse model). Secondly, we evaluated whether overlap among ranked gene lists was statistically significant (Figure 4) [32]. Human transcripts were ranked according to the estimated fold-change difference between lesional and normal skin, and likewise for each mouse model, transcripts were ranked according to the estimated fold-change difference between psoriasiform and normal skin. This analysis revealed that ranked transcript lists associated with human psoriasis significantly overlapped with corresponding lists associated with each psoriasiform phenotype, regardless of whether the top *N* psoriasis-increased or psoriasis-decreased human transcripts were considered (P<0.05; 10≤ *N* ≤5000; see Figure 4). These conclusions were further supported by three additional statistical methods, including analysis of adjusted residuals (Figure S1) [33], “detection rate” ROC curves (Figure S2) [34], and the gene set enrichment score statistic proposed by Subramanian et al. [35] (i.e., “GSEA analysis”, see Figure S3). We note that, for psoriasis-decreased genes, GSEA-generated p-values were non-significant with respect to the K5-TGFβ1 model (P = 0.318; Figure S3).

**Figure 5:**
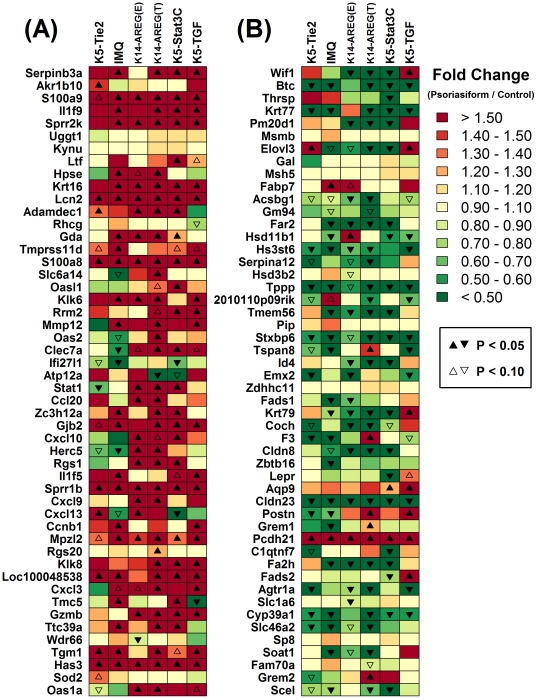
Trademark gene expression patterns of human psoriasis and expression of orthologous genes in mouse psoriasiform phenotypes. Genome wide expression data from human psoriatic skin samples (*n* = 58 patients) and normal skin (*n* = 64 subjects) was analyzed to identify (A) the 50 genes most strongly increased in human psoriasis and (B) the 50 genes most strongly decreased in human psoriasis (both lists exclude any psoriasis-increased or psoriasis-decreased human gene that lacks an orthologous mouse gene). For each human gene, a matching transcript associated with an orthologous mouse gene was identified, and the expression of this mouse transcript was compared in psoriasiform (*n* = 3) and normal skin (*n* = 3). In part (A), mouse genes are listed in descending order according to the fold-change estimate calculated with respect to the human orthologue (psoriasis/control), such that transcripts orthologous to genes most strongly *increased* in human psoriasis are positioned near the top of the figure. In part (B), mouse genes are listed in ascending order according to the fold-change estimate calculated with respect to the human orthologue (psoriasis/control), such that transcripts orthologous to genes most strongly *decreased* in human psoriasis are positioned near the top of the figure. The colors in (A) and (B) correspond to the observed fold-change difference between expression in psoriasiform mouse skin and normal skin obtained from control mice, with red indicating elevated expression in psoriasiform skin and green indicating decreased expression (see scale). Filled up-triangles denote transcripts with significantly increased expression in psoriasiform mouse skin (P<0.05) and filled down-triangles denote transcripts with significantly decreased expression in psoriasiform mouse skin (P<0.05). Unfilled up or down triangles denote transcripts for which the expression difference between psoriasiform and control mouse skin was marginally significant (0.05≤ P <0.10).

Correspondence between human psoriasis and mouse phenotypes was also evident from inspection of the “trademark” expression patterns of human psoriasis, which we identified as the most consistent and pronounced gene expression differences between psoriatic plaques and normal human skin (Figure 5). In all five mouse phenotypes, there was increased expression of *S100a9*, *Lcn2*, *S100a8*, *Sprr1b*, *Mpzl2*, *Has3* (Figure 5A), as well as decreased expression of *Tppp*, *Stxbp6*, and *Cldn23* (Figure 5B), and each of these effects is consistent with characteristic expression patterns in clinical psoriasis. Additionally, we identified a set of 27 mouse genes (with human orthologues) that were significantly increased in all five mouse phenotypes (P<0.05), and in 23 of these cases, the orthologous human gene was elevated in skin from psoriasis patients (Figure S4). Likewise, we identified a set of 44 mouse genes (with human orthologues) that were significantly decreased in all five mouse phenotypes (P<0.05), and in 32 of 44 cases, associated human genes were correspondingly decreased in human psoriasis (Figure S5). There were also examples in which psoriasiform phenotypes failed to recapitulate a robust gene expression indicator of human psoriasis. For instance, expression of protocadherin 21 (*PCDH21*) is decreased by 68.4% in human psoriasis (FDR-adjusted P  = 6.62×10^−37^), but the mouse ortholog *Pcdh21* was in fact elevated 1.5-fold in K5-Tie2 lesions (P = 0.025), 54.2-fold in IMQ lesions (P = 2.43×10^−7^), 5.2-fold in K14-AREG ear lesions (P = 1.1×10^−3^), 24.9-fold in K14-AREG tail lesions (P = 1.48×10^−6^), 7.4-fold in K5-Stat3C lesions (P = 3.73×10^−6^), and 22.5-fold in K5-TGFβ1 lesions (P = 4.8×10^−4^) (Figure 5B and Figure S4). Another robust feature of human psoriasis was increased expression of C-type lectin domain family 7 member A (*CLEC7A*/*DECTIN-1*), which encodes a membrane receptor that mediates Th1/Th17 immune responses [36]. In human psoriasis, expression of *CLEC7A* was elevated 8.7-fold (FDR-adjusted P<1.59×10^−58^), but expression of the mouse ortholog *Clec7a* was not altered in the K5-Tie2 phenotype (P = 0.843) and was decreased by 43% in the IMQ phenotype (P = 0.046) (Figure 5A).

### Each mouse phenotype exhibits a shift in epidermis- and keratinization- associated expression patterns that parallels clinical psoriasis

**Figure 6:**
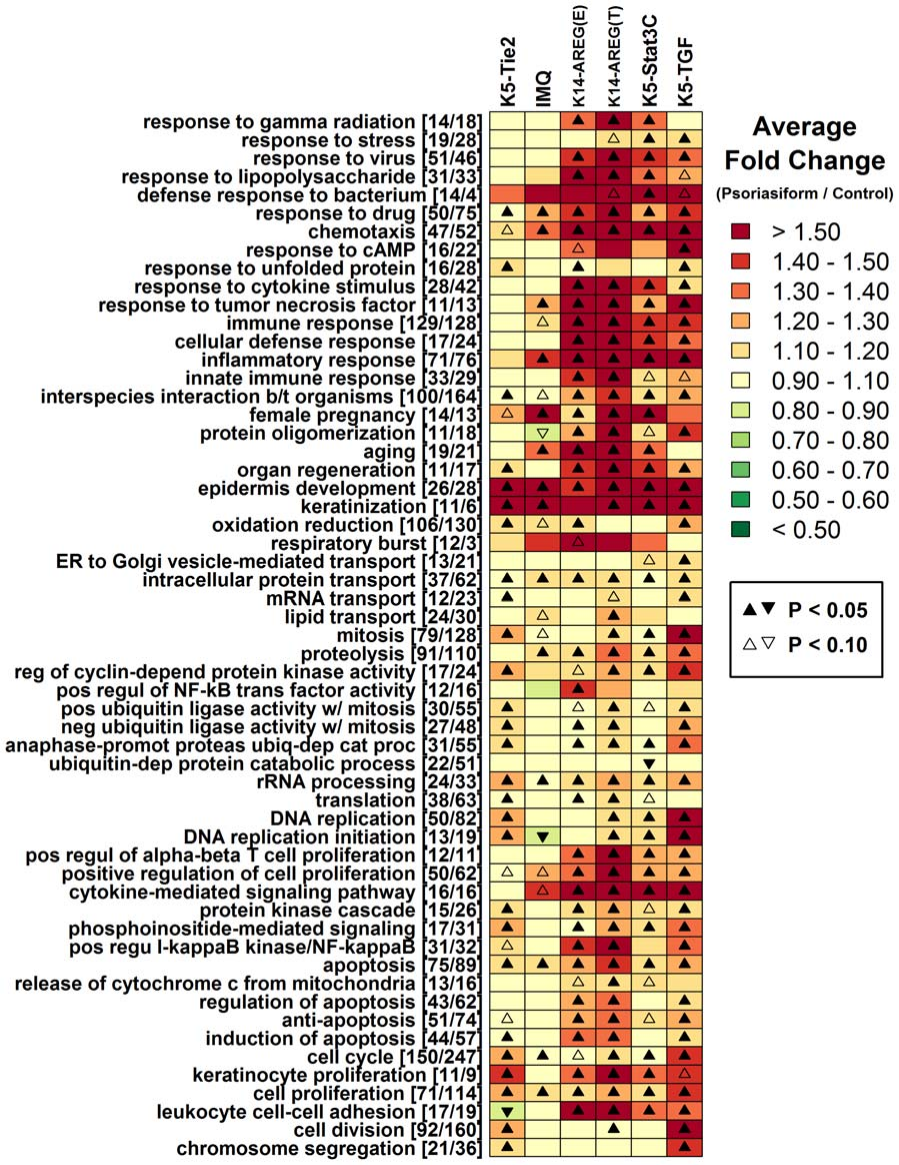
Gene ontology biological processes overrepresented among genes exhibiting increased expression in human psoriasis. A total of 2617 transcripts were identified as significantly elevated in psoriatic plaques obtained from human patients relative to normal skin obtained from control subjects (FDR-adjusted P<0.05 and log_2_-transformed fold-change greater than 0.50). These psoriasis-increased transcripts were analyzed to identify gene ontology biological process terms significantly over-represented (P<0.05). For each over-represented term, we determined which of the 2617 transcripts were annotated with the term, and for these human transcripts, we identified a set of mouse transcripts associated with orthologous genes. For each mouse transcript within this set, we calculated the expression ratio between psoriasiform and normal mouse skin, and determined the average value of this ratio among all transcripts in the set. The analysis was repeated with respect to each of the mouse skin phenotypes. Colors in the chart reflect the average fold-change ratio (psoriasiform/control) of the set of mouse transcripts associated with the gene ontology term listed in each row (see scale). Triangle symbols indicate whether the average fold-change estimate associated with mouse transcripts is significantly different from one (see legend; two-sample t-test). The number of transcripts associated with each GO term is indicated in brackets (e.g., [*x*/*y*], where *x* denotes the number of human transcripts and *y* represents the number of mouse transcripts derived from orthologous genes). Since mouse transcripts included in this analysis were orthologous to genes exhibiting *elevated* expression in human psoriasis, correspondence between human psoriasis and mouse phenotypes is indicated by red colors and up-triangle symbols.

**Figure 7:**
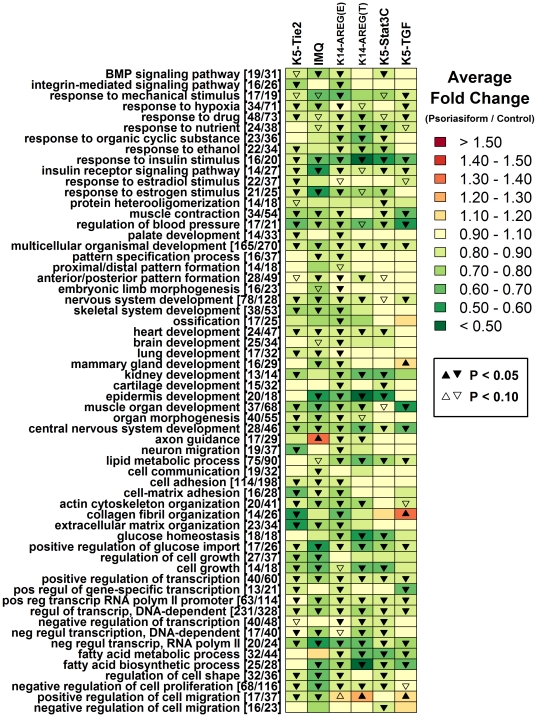
Gene ontology biological processes overrepresented among genes exhibiting decreased expression in human psoriasis. A total of 3540 transcripts were identified as significantly decreased in psoriatic plaques obtained from human patients relative to normal skin obtained from control subjects (FDR-adjusted P<0.05 and log_2_-transformed fold-change less than -0.50). These psoriasis-decreased transcripts were analyzed to identify gene ontology biological process terms significantly over-represented (P<0.05). For each over-represented term, we determined which of the 3540 transcripts were annotated with the term, and for these human transcripts, we identified a set of mouse transcripts associated with orthologous genes. For each mouse transcript within this set, we calculated the expression ratio between psoriasiform and normal mouse skin, and determined the average value of this ratio among all transcripts in the set. The analysis was repeated with respect to the K5-Tie2 phenotype, IMQ phenotype, K14-AREG phenotype on ear skin, K14-AREG phenotype on tail skin, K5-Stat3C phenotype, and K5-TGFβ1 phenotype. Colors in the chart reflect the average fold-change ratio (psoriasiform/control) of the set of mouse transcripts associated with the gene ontology term listed in each row (see scale). Triangle symbols indicate whether the average fold-change estimate associated with mouse transcripts is significantly different from one (see legend; two-sample t-test). The number of transcripts associated with each GO term is indicated in brackets (e.g., [*x*/*y*], where *x* denotes the number of human transcripts and *y* represents the number of mouse transcripts derived from orthologous genes). Since mouse transcripts included in this analysis were orthologous to genes exhibiting *decreased* expression in human psoriasis, correspondence between human psoriasis and mouse phenotypes is indicated by green colors and down-triangle symbols.

Gene Ontology (GO) biological processes significantly overrepresented among transcripts increased or decreased in human psoriasis were identified (P<0.05), representing the most salient features of the human psoriasis gene expression signature (Figures 6 and 7). For each overrepresented process, we identified the associated human genes altered in clinical psoriasis, and determined whether orthologous mouse genes were correspondingly altered in mouse psoriasiform phenotypes. This analysis revealed that many key aspects of the human psoriasis gene expression signature were mirrored by mouse psoriasiform phenotypes. The most striking point of correspondence, shared among the five mouse models, was elevated expression of transcripts involved in epidermal development and keratinization (see Figure 6). For instance, among transcripts increased significantly in human psoriasis, we identified 26 transcripts associated with the “epidermis development” (GO:0008544) gene ontology term (e.g., *KRT16*, *KRT17*, *ELF3*). Based on the human-mouse orthology, there were 28 mouse transcripts associated with these human transcripts, and of these, 92.9% were elevated in K5-Tie2 (26 of 28; P = 1.08×10^−7^), 89.3% in IMQ (25 of 28; P = 1.52×10^−6^), 71.4% in K14-AREG ear (20 of 28; P = 6.27×10^−3^), 92.9% in K14-AREG tail (26 of 28; P = 1.08×10^−7^), 75% in K5-Stat3C (21 of 28; P = 1.86×10^−3^), and 82.1% in K5-TGFβ1 (23 of 28; P = 9.00×10^−5^). Among transcripts decreased in human psoriasis, there was even stronger similarity among mouse models, involving a broad range of biological processes for which expression patterns were correspondent between psoriasiform phenotypes and human (e.g., response to insulin stimulus, development, transcription; see Figure 7).

These analyses were repeated based upon the KEGG pathways overrepresented among transcripts increased or decreased in human psoriasis (Figures S6 and S7). As an alternative analysis method, we also extracted sets of human-mouse gene pairs with correspondent expression shifts in mouse phenotypes and human psoriasis, and identified GO biological process terms overrepresented within these gene sets. This strategy identified gene categories that, while not necessarily the most salient features of the human psoriasis expression signature, were nonetheless descriptive of points at which human psoriasis and mouse phenotypes correspond (Figure S8 and ).

### The mouse phenotypes diverge with respect to immune-associated gene expression patterns and mitosis-related transcription

**Figure 8:**
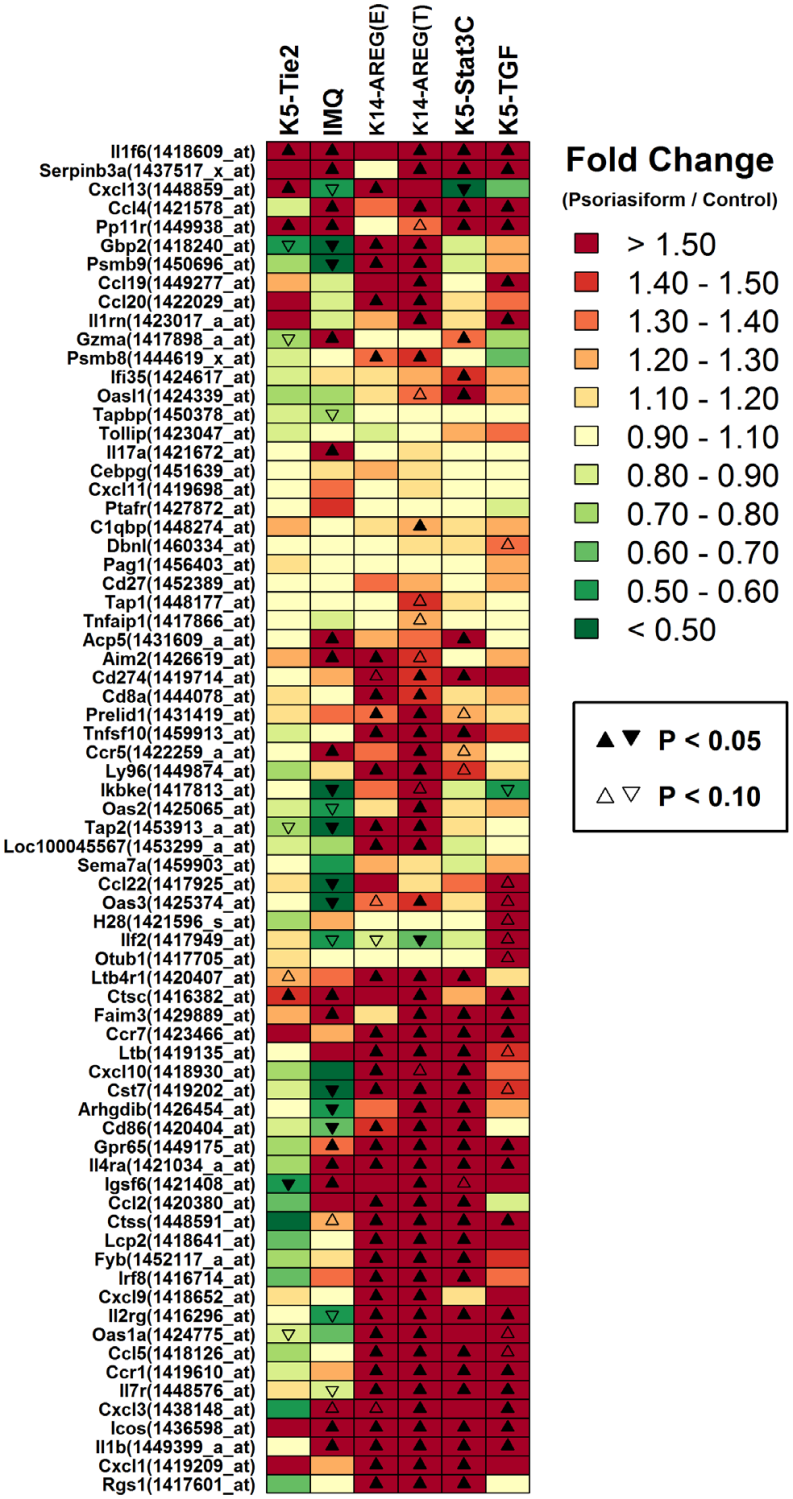
Mouse orthologs of immune response genes increased in human psoriasis. A total of 129 transcripts associated with “immune response” were significantly elevated in human psoriasis (GO:0006966). With respect to these 129 psoriasis-increased transcripts, we identified 128 corresponding mouse transcripts derived from orthologous mouse genes. A subset of these 128 transcripts is listed in the figure (left margin). The heat map image describes the response patterns of these transcripts in psoriasiform phenotypes relative to normal skin in control mice. Red colors correspond to increased expression in psoriasiform phenotypes and green colors correspond to decreased expression (see scale; right margin). Up- and down-triangles denote transcripts for which the fold-change difference between lesion and normal mouse skin is significant or marginally significant. Since listed transcripts are orthologous to human genes exhibiting increased expression in clinical psoriasis, correspondence to the human disease is denoted by red colors (i.e., increased expression in psoriasiform phenotypes).

While key features of human psoriasis were recapitulated by mouse model phenotypes, points of non-correspondence relative to each other and to human psoriasis were also identified. Most notably, there was disparity between the K5-Tie2 and IMQ phenotypes relative to other mouse models, with respect to psoriasis-increased genes with immune-associated gene ontology terms, such as response to virus, response to lipopolysaccharide, response to cytokine stimulus, cellular defense response, innate immune response and positive regulation of αβ T-cell proliferation (Figures 6 and 8). This disparity between K5-Tie2 and IMQ phenotypes relative to other mouse models was also evident based upon inspection of KEGG pathway terms associated with transcripts elevated in human psoriasis (e.g., primary immunodeficiency, natural killer cell mediated cytotoxicity, toll-like receptor signaling pathway, cytokine-cytokine receptor interaction and leukocyte adhesion; see Figure S6). This trend was further illustrated by GO analysis of human-mouse transcript pairs, with conflicting expression patterns in clinical psoriasis and mouse phenotypes (Figures S10 and S11). This analysis revealed that pairs involving a psoriasis-increased transcript in humans and an orthologous transcript decreased in K5-Tie2 or IMQ lesions were often associated with immunity (e.g., response to virus, innate immune response; see Figure S10). The overall pattern is well-illustrated by the 129 transcripts increased in human psoriasis that were associated with the “immune response” (GO:0006955) GO term (see Figure 8). Of the 128 mouse transcripts that could be matched to the 129 psoriasis-upregulated human transcripts mapping to this term, more than 77% (>99/128) were correspondingly elevated in the K14-AREG, K5-Stat3C and K5-TGFβ1 phenotypes, but only 44% (56/128) and 59% (75/128) were correspondingly elevated in the K5-Tie2 and IMQ phenotypes. These observations suggest that, as compared to the IMQ and K5-Tie2 models, the K14-AREG, K5-Stat3C and K5-TGFβ1 phenotypes better recapitulate immune-associated gene expression patterns characteristic of clinical psoriasis.

Human psoriasis is associated with increased expression of transcripts involved in cellular proliferation and KC differentiation and this was reflected in each mouse gene expression phenotype (Figure 6). However, IMQ-treated mice were less correspondent with this aspect of the human psoriasis expression pattern, while the K5-Tie2 and K5-TGFβ1 models were most similar to the clinical disease in this respect (e.g., see mitosis, DNA replication, DNA replication initiation, KC proliferation, cell division from Figure 6; see cell cycle and DNA replication from Figure S6; see mitotic anaphase from Figure S10). This trend is exemplified by 128 transcripts associated with mouse genes orthologous to the human “mitosis” genes with elevated expression in human psoriasis (e.g., GO:0007067; see Figure S12). Among these transcripts, more than 87% are increased in the K5-Tie2 and K5-TGFβ1 phenotypes (≥112 of 128), while 60.2% (77/128), 43.7% (56/128), 69.5% (89/128) and 64.1% (82/128) are increased in the IMQ, K14-AREG (ear), K14-AREG (tail) and K5-Stat3C phenotypes, respectively.

### The K14-AREG, K5-Stat3C and K5-TGFβ1 phenotypes exhibit a heightened inflammation signature relative to that of K5-Tie2 and IMQ psoriasiform lesions

**Figure 9:**
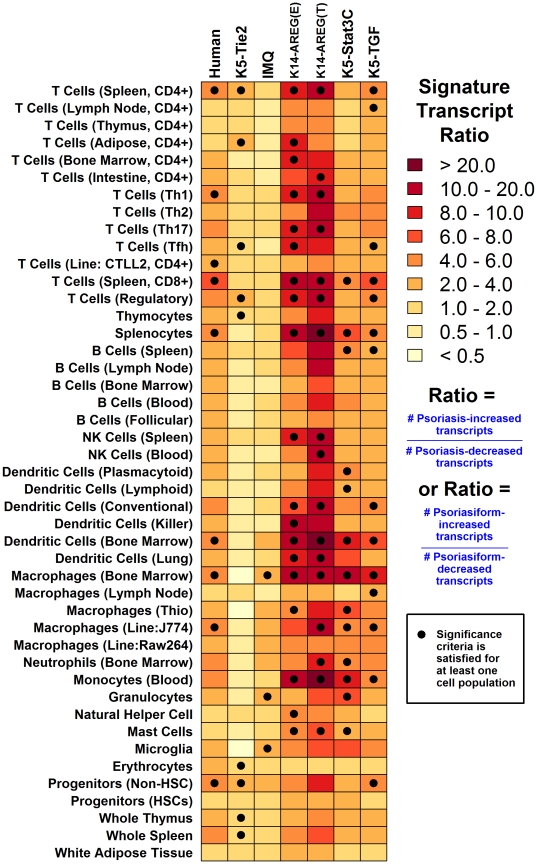
Leukocyte infiltration signatures of human psoriasis and mouse psoriasiform lesions. The immunophenotyping algorithm developed by Swindell et al. (31) was used to generate inflammation profiles for human psoriasis and the K5-Tie2, IMQ, K14-AREG, K5-Stat3C and K5-TGFβ1 psoriasiform lesions. Signature transcripts highly expressed within different cell populations were identified, where most cell populations were leukocyte subsets isolated from mice (e.g., T-cells, B cells, macrophages; see Methods for details). For each set of *n* signature transcripts associated with a given cell population, and for each psoriasiform phenotype, we identified the number of transcripts with increased expression in mouse psoriasiform lesions (*n*_1_) and the number of transcripts with decreased expression in mouse psoriasiform lesions (*n*_2_). For each set and each mouse phenotype, the ratio of psoriasiform-increased to psorisiform-decreased transcripts was calculated (i.e., ratio  =  *n*_1_/*n*_2_, where *n*_1_ + *n*_2_  =  *n*). Colors in the chart corresponds to this calculated ratio (see scale), where darker red colors indicate that the signature transcripts of cell populations (rows) tended to be elevated in the mouse psoriasiform phenotype (columns). Filled symbols are used to indicate cell populations for which the proportion of signature transcripts elevated in psoriasiform phenotypes (i.e., the *n*_1_/*n*_2_ ratio) was significantly large (see Methods for significance criteria). Darker colors (larger *n*_1_/*n*_2_ ratios) thus indicate cell populations that, based upon the observed gene expression patterns, appear likely to comprise the inflammatory infiltrate associated with a given mouse psoriasiform phenotype. In the first column, a similar methodology was applied with respect to human psoriasis. However, for each cell population, we identified human transcripts associated with genes orthologous to the *n* signature transcripts, and among these human transcripts, we evaluated the ratio of psoriasis-increased to psoriasis-decreased transcripts.

We identified strong differences among mouse phenotypes with respect to the immune-associated gene expression patterns characteristic of human psoriasis, with the K14-AREG, K5-Stat3C and K5-TGFβ1 phenotypes exhibiting a closer correspondences to human psoriasis (Figure 8). One possibility is that models differ in terms of the type and abundance of leukocytes present within psoriasiform plaques, leading to dissimilar expression patterns among genes annotated with immune-related GO terms or KEGG pathways. To evaluate this possibility, we characterized each mouse phenotype using a microarray-based immunophenotyping algorithm, which estimates overall inflammation intensity and also identifies leukocyte subsets underlying an inflammation signature within microarray data (Figure 9) [31] (see also Haider et al. [37] for a similar approach). In brief, the algorithm utilizes gene expression profiles of cell populations harvested from mouse tissues (mostly leukocytes; e.g., T-cells, DCs, macrophages), and for each population, a set of “signature transcripts” is identified, which consists of transcripts highly expressed in that population relative to normal mouse skin. If a given leukocyte population is part of the infiltrate in lesional skin, it is expected that signature transcripts associated with that population will be disproportionately elevated in lesional skin relative to normal skin from control mice, and this information is used to establish an “inflammation profile” [31]. In the present context, we have also adapted this approach to estimate an inflammation profile for human psoriasis, based upon expression patterns of human genes orthologous to signature transcripts associated with individual mouse leukocyte populations (Figure 9).

The immunophenotype of human psoriatic plaques was consistent with elevated abundance of Th1 T-cell, CD4+ T-cell, CD8+ T-cell, DC and macrophage signatures (P<0.05; see Figure 9), which was an expected based upon prior clinical studies of psoriatic lesions [38]. Most of these trends, however, were absent from the IMQ phenotype, and in general, the intensity of inflammation was low in IMQ-generated lesions, with significant evidence for invasion by macrophages and granulocytes (P<0.05), but no other leukocyte subsets (Figure 9). To some degree, inflammation intensity was also relatively low in lesions from K5-Tie2 mice, and surprisingly, there was no significant evidence for macrophage or DC infiltration, although there was evidence for infiltration by CD4+ and regulatory T-cells (P<0.05; Figure 9). Inflammation intensity was strongest in the K14-AREG, K5-Stat3C and K5-TGFβ1 models, with the strongest patterns associated with K14-AREG lesions (ear and tail), for which there was significant evidence of infiltration by CD4+ T-cells, Th1 T-cells, Th17 T-cells, CD8+ T-cells, regulatory T-cells, NK cells, DCs, macrophages and monocytes (P<0.05; Figure 9). The immunophenotypes of K5-Stat3C and K5-TGFβ1 models were comparable in most respects, although for K5-TGFβ1 lesions, there was stronger indication of T-cell infiltration, with significant CD4+ T-cell, CD8+ T-cell, helper T-cell and regulatory T-cell signatures (P<0.05; Figure 9). Taken together, these analyses support a ranking of mouse phenotypes with regard to overall inflammation intensity, with the strongest inflammation signature in K14-AREG lesions, moderately strong inflammation in K5-Stat3C and K5-TGFβ1 lesions, weak-to-moderate inflammation in K5-Tie2 lesions, and weak inflammation in the phenotype generated by topical application of IMQ.

### The K14-AREG, K5-Stat3C and K5-TGFβ1 lesions exhibit stronger increases in TNF-α, IFN-γ and IL-6 expression with heightened TNF-α and IFN-γ-dependent KC responses

The above results indicated that mouse models differed with respect to immune-associated gene expression patterns (Figures 6 and S6) and also with respect to their microarray-based inflammation profiles (Figure 9). We therefore evaluated the expression of selected cytokines and chemokines thought to contribute to initiation or maintenance of the inflammatory cascade in human psoriasis (e.g., *TNF*, *IL22*, *IL6*, *CXCL1*) (Figure S13). This analysis pointed to key expression responses that were more prominent in the K14-AREG (ear and tail), K5-Stat3C and K5-TGFβ1 models as compared to the K5-Tie2 and IMQ models, such as increased expression of tumor necrosis factor-α (*Tnf*), interferon (IFN)-γ (*Ifng*), interleukin (IL)-6 (*Il6*) CXC-chemokine ligand 10 (*Cxcl10*), and integrin β2 (*Itgb2*) (Figure S13). For instance, we noted little change in *Tnf* expression for K5-Tie2 or IMQ lesions (6% increase for K5-Tie2 and 13% decrease for IMQ; P>0.43), while expression of *Tnf* was elevated 31% in K14-AREG ear lesions (P = 0.063), 48% in K14-AREG tail lesions (P = 0.047), 34% in K5-Stat3C lesions (P = 0.042) and 116% in K5-TGFβ1 lesions (P = 0.01). These results are consistent with decreased accumulation of key cytokines in psoriasiform lesions of K5-Tie2 and IMQ mice, which may account for lower intensity inflammation within these models (Figure 9).

**Figure 10:**
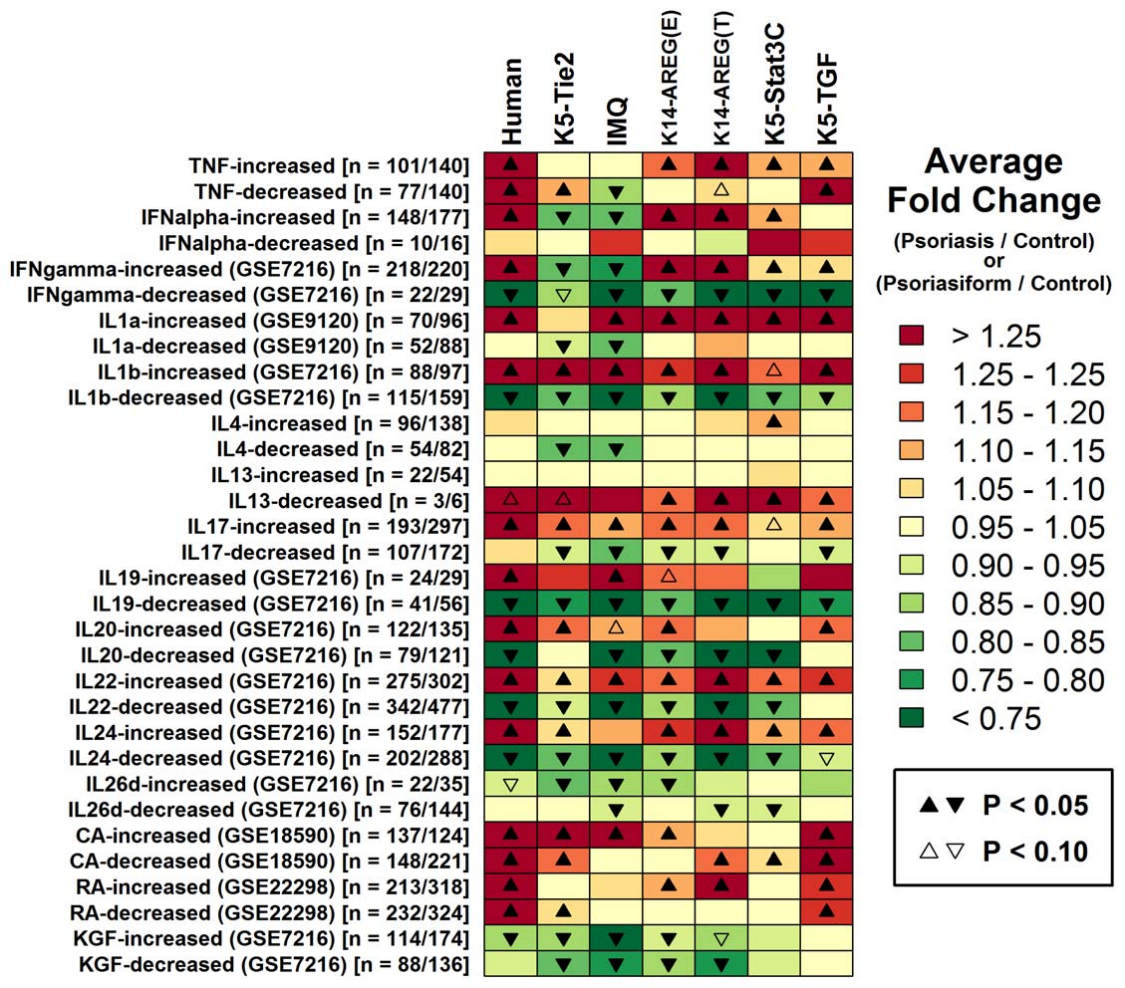
Gene expression signatures associated with cytokine stimulation and differentiation of keratinocytes (KCs). Microarray data was used to identify human transcripts exhibiting increased or decreased expression in KCs treated with cytokines (e.g., TNF, IFN-α, IFN-γ), agents that induce differentiation (calcium (CA), KC growth factor (KGF)), and agents that inhibit differentiation (retinoic acid (RA)). Each row in the chart corresponds to a set of transcripts, with the number of human and associated mouse transcripts indicated in brackets (*n*  =  *x*/*y* denotes *x* human transcripts and *y* mouse transcripts associated with orthologous mouse genes). For human transcripts in each set, the average expression ratio between psoriasis and normal skin samples (psoriasis/normal) was calculated, and the average ratio obtained for each set is denoted in the first column according to the color code described in the legend. Likewise, for mouse transcripts within each set, the average expression ratio between psoriasiform and normal skin (psoriasiform/normal) was calculated with respect to each mouse model, and the average ratio value for each set and mouse model is indicated according to the color code (see legend). Triangle symbols indicate whether, on average, fold change estimates differ significantly from one (see legend; two-tailed t-test). In some cases (rows), gene sets were defined based upon data from Gene Expression Omnibus (GEO) and the GEO series identifier is given in parentheses (e.g., GSE7216).

Gene expression shifts in both human psoriasis and mouse psoriasiform phenotypes are, in part, a consequence of KC responses to the local cytokine and chemokine environment. While mouse phenotypes differed with respect to localized abundance of mRNAs encoding key cytokines and chemokines (Figure S13), these disparities at the RNA level may or may not influence steady-state cytokine abundance or KC responses. We therefore identified sets of genes associated with such *in vitro* KC responses to cytokine treatment and evaluated how these gene sets were altered in both psoriatic skin from humans and lesional skin from mouse model phenotypes (Figure 10). This indicated that K14-AREG (ear), K14-AREG (tail), K5-Stat3C and K5-TGFβ1 phenotypes exhibited increased expression of transcripts that are induced by *in vitro* treatment of KCs with TNF-α, IFN-α and IFN-γ, while such responses were not observed with respect to the K5-Tie2 and IMQ phenotypes (Figure 10). Additionally, as transcriptional markers of KC differentiation, we identified gene sets associated with calcium supplementation of cultured KCs, and these gene sets were more strongly elevated in K5-Tie2 and K5-TGFβ1 lesions (Figure 10).

## Discussion

The development of a “high-fidelity” mouse model of psoriasis would provide a valuable experimental system for investigating mechanisms of psoriasis pathogenesis and anti-psoriatic therapies. Despite differences that exist between humans and mice, mouse disease models have made important contributions to the investigation of major disease processes, including for example, heart disease [39], Alzheimer's disease [40], diabetes [41], cancer [42], as well as autoimmune syndromes such as lupus erythematosus and rheumatoid arthritis [43], [44]. Because the primary disease symptoms of psoriasis occur at the skin surface, direct comparisons can be made between affected skin from patients and putative mouse models. We have followed this approach and have applied functional genomics methods to compare global transcriptional signatures of psoriasis phenotypes in humans and five psoriasis mouse models. With respect to an unbiased choice of psoriasis-associated GO and KEGG pathway terms, we identified specific similarities and differences between human psoriasis and each mouse model, and we suggest that these differences can be translated into strengths and weaknesses of each mouse model. For each model, gene expression patterns associated with epidermis development and keratinization mirrored those of clinical psoriasis. However, we noted divergence among models with respect to immune-associated gene expression, with a heightened inflammatory signature in lesions from K14-AREG, K5-Stat3C and K5-TGFβ1 mice, along with stronger elevation of mRNAs coding key cytokines and chemokines (e.g., *Tnf*, *Ifng*, *IL-6*) and more pronounced TNF-α and IFN-γ-driven expression patterns. Nevertheless, at a global level, there is strong and statistically significant similarity between expression patterns in clinical psoriasis and those of each mouse model evaluated by our study.

Psoriasis results from an interaction between activated immunocytes and KCs, in which KCs exhibit abnormal maturation and proliferation in response to a complex cytokine network that mediates disease maintenance and progression [38]. The key role of the immune response and inflammatory process has been convincingly supported by the identification of major histocompatibility complex and cytokine-associated loci in genome-wide association analyses [45], the effectiveness of immunosuppressant therapies in treatment of the disease [46], and the observation that psoriasis can be transferred between donor and recipient with bone marrow transplantation [47]. For these reasons, a consensus point among investigators is that a realistic psoriasis mouse model should exhibit an inflammatory infiltrate resembling that found in clinical psoriasis, including T-cells, DCs and neutrophils, and that the psoriasiform phenotype should be both T-cell dependent and responsive to drug treatments targeting the immune system [3]–[5]. It was therefore significant, in the present study, to observe that gene expression signatures of mouse psoriasiform phenotypes diverged with respect to immune-associated transcripts, with three phenotypes more closely mirroring clinical psoriasis (i.e., K14-AREG, K5-Stat3C and K5-TGFβ1) as compared to two others (i.e., K5-Tie2 and IMQ). This disparity likely involves differences in the relative abundance of certain immune cell subsets within lesional skin of psoriasiform phenotypes, which would have consequent effects on the cytokine and chemokine milieu and development of the inflammatory cascade. For instance, the K14-AREG, K5-Stat3C and K5-TGFβ1 phenotypes showed a stronger inflammation signature overall, with significant evidence for infiltration by T-cells, monocytes, DC and macrophages (sure 9). These phenotypes contrast with that of the K5-Tie2 model, where our immunophenotyping algorithm supported increased expression of transcripts associated with CD4+ T-cells, but did not provide evidence for increased expression of transcripts highly expressed in cells from the monocyte-DC/macrophage lineage (Figure 9). Similarly, for IMQ-generated lesions, evidence of macrophage infiltration was obtained, but there was little or no indication of T-cell infiltration (Figure 9). Previously, immunohistochemical methods have detected increased abundance of CD8+ T cells, F4/80+ macrophages and CD11b and CD11c+ myeloid cells in K5-Tie2 lesions [12], [48], as well as increased abundance of CD4+ and CD8+ T-cells in IMQ-generated lesions [13]. In these cases, however, the number of infiltrating immunocytes may not be sufficient to have a discernable impact on gene expression patterns, and this is likely to at least partially account for the observed disparity among models with respect to immune-associated transcripts (Figure 8).

The environment of cytokines and chemokines in mouse psoriasiform lesions is largely shaped by early-stage inflammation responses. Along these lines, mouse phenotypes also differed in their relative expression level of the proinflammatory cytokines TNF-α, IFN-γ and IL-6, where in each case expression was more strongly elevated in K14-AREG, K5-Stat3C and K5-TGFβ1 lesions as compared to K5-Tie2 or IMQ lesions (Figure S13). Moreover, *in vitro* KC transcriptional responses to TNF-α, IFN-γ and IFN-α stimulation were not discernable with respect to the K5-Tie2 or IMQ expression signatures, but were discernable with respect to K14-AREG, K5-Stat3C and K5-TGFβ1 expression signatures (Figure 10). TNF-α is generated by NK-T and αβ-T-cells, macrophages and KCs, and is known to reinforce inflammatory processes. In psoriasis, the pathogenic role of TNF-α has been demonstrated by the effective treatment of patients with anti-TNF therapies [49]. TNF-α is also a key component of local cytokine networks, and indeed, the quantitatively lower increase in TNF-α expression that we observed in K5-Tie2 and IMQ-generated lesions may explain the similar trends observed for IFN-γ and IL-6. In particular, a previous study using peripheral blood mononuclear cells has shown that TNF blockade suppresses IFN-γ expression, while in turn, IFN-γ blockade suppresses IL-6 expression [50]. Our findings thus suggest that psoriasis mouse models can be differentiated with respect to a TNF-α/IFN-γ/IL-6 axis, which ultimately, may be reflective of disparities in the abundance of certain TNF-generating immunocytes (e.g., macrophages, DCs or T-cells).

KC proliferation and consequent hyperplasia of the epidermal layer is a hallmark feature of clinical psoriasis [38]. It is therefore noteworthy that our analysis detected differences among models related to mitosis-associated gene expression patterns. Mitosis-associated transcripts were most strongly elevated in K5-TGFβ1 lesions, and a comparable trend was evident for K5-Tie2 lesions, and in this regard, the K5-TGFβ1 and K5-Tie2 models were generally most similar to the mitotic gene expression signature of clinical psoriasis (see Figure S12). This trend was supported, for example, by expression patterns of many transcripts involved in DNA replication and the transitions between mitotic phases, including cyclin A2 (*Ccna2*), cyclin F (*Ccnf*) and budding uninhibited by benzimidazoles 1 homolog (*Bub1b*) (Figure S12).

The psoriasis phenotypes evaluated in this study were generated on outbred, inbred and hybrid genetic backgrounds, including outbred CD1 mice (K5-Tie2), the inbred strain C57BL/6 (IMQ), as well as hybrid FVB/NCrIBR (K14-AREG), FVB/NHsd (K5-Stat3C), and ICR/B6D2 mice (K5-TGFβ1). While our analysis has identified disparities among models, it is possible that these disparities are, in part, driven by characteristics of individual mouse strains, which might in turn modulate the direct effects of manipulations we have considered. For this reason, we suggest that any psoriasis mouse model should be considered jointly with the genetic background with which it is associated (e.g., the “K5-Tie2/CD1 model”, the “B6/IMQ model”, etc.). Along these lines, an important avenue for future investigation is to evaluate background-dependence of manipulations that give rise to psoriasis-like phenotypes in mouse.

The approach used in this study to evaluate psoriasis mouse models represents a general strategy that can be applied to score alternative mouse phenotypes with resemblance to specific human diseases. Potentially, the same methodology could be applied to determine whether psoriasiform phenotypes we evaluated are equally or better-suited as models for other inflammatory skin conditions, such as atopic dermatitis or allergic contact dermatitis. The microarray-based analytical approach we have implemented does not replace conventional validation criteria for psoriasis mouse models, but extends these criteria by providing a quantitative “yardstick” that can be used to clearly judge progress towards more realistic mouse phenotypes. This can guide development of new psoriasis mouse models by identifying combinations of genetic manipulations that complement each other well and also provide guidance to scientists in choosing the most appropriate mouse model to use when testing specific mechanistic and therapeutic hypotheses. Ultimately, this may lead to development of psoriasis mouse models with stronger biochemical similarity to the human disease.

## Materials and Methods

### Ethics Statement

This study was conducted in compliance with good clinical practice and according to the Declaration of Helsinki principles. Informed written consent was obtained from all human subjects, under protocols approved by the institutional review board of the University of Michigan (HUM00037994). All animal protocols were approved by animal welfare committees at each participating institution; Case Western Reserve University Institutional Animal Care and Use Committee (IACUC, #2009-0193), Erasmus Medical Center Animal Ethics Committee (Advies DEC Nr. EUR 1846 (EMCnr. 128-09-09), Institutional Animal Care and Use Committee of the University of Texas, Austin (AUP-2010-00029), Institutional Animal Care and Use Committee of the University of Colorado (B-850008(08)2E and the Institutional Animal Care and Use Committee of Mayo Clinic (A1009-Rochester).

### Human and Mouse Expression Data

The patient population and sample processing methods for the collection of human microarray data has been described in a recent report [51]. In brief, the study involved 58 psoriasis patients (ages 21- 69) and 64 normal healthy control subjects (ages 18 - 45) recruited from areas surrounding Detroit, Michigan. The 58 psoriasis patients were chosen on the basis of having one or more psoriatic plaques not limited to the scalp region. If the patient had only one plaque, the single plaque was large in size (greater than 1% of total body area). No systemic anti-psoriatic treatments were used for 2 weeks prior to biopsy, and no topical treatments were used for 1 week prior to biopsy. In control subjects, biopsies were always taken from the buttocks or upper thighs. RNA samples were hybridized to the Affymetrix Human Genome U133 Plus 2.0 Array, which includes probesets corresponding to 54675 human transcripts. All the microarray data are MIAME compliant. Raw microarray data from the psoriasis cohort has been deposited in the NCBI Gene Expression Omnibus (GEO, http://www.ncbi.nlm.nih.gov/geo) and is accessible through GEO Series accession number GSE13355.

Generation of transgenic mice bearing psoriasiform phenotypes has been described in previous reports [12]–[16]. The K5-Tie2 mice were generated by crossing a KC-specific K5-tTA driver line *(Tg(KRT5-tTA)1216Glk)* with a Tet^OS^Tek/Tie2 responder line (*Tg(TetOS-Tek)1Dmt*) that had been generated on an outbred CD1 background [52], [53]. The generation of K14-AREG (FVB/NCrIBR-*Tg(KRT14-AREG)3Pwc*), K5-Stat3C (FVB-Tg(KRT5-Stat3*A661C*N663C)1Jdg) and K5-TGFb1 ((ICRxB6D2)F1-*Tg(KRT5-TGF-B1)F2020Xjw*) transgenic mice is described in the reports of Cook et al. [14], Sano et al. [15] and Li et al. [16], respectively. For all experiments involving transgenic mice, experimental and control animals were genotyped at weaning using PCR, and development of psoriasiform lesions was spontaneous and not induced by wounding (e.g., tape stripping). For the IMQ-generated phenotype, the treatment protocol was to apply a daily topical dose of 62.5 mg IMQ cream (5% Aldara; 3M Pharmaceuticals) to the shaved back region of 8–10 week old C57BL/6 mice. This treatment was carried out for six consecutive days, and during this time, control animals were treated with a vehicle cream (Vaseline Lanette cream; Fagron). In all experiments, adult mice were euthanized at 10–20 weeks of age at which time ear, tail or back skin samples were flash frozen and stored at −80°C prior to the isolation of total RNA. We have evaluated back skin samples from the K5-Tie2, IMQ, K5-Stat3C and K5-TGFβ1. In K14-AREG mice, however, we have focused on tail and ear skin phenotypes to be consistent with previous studies [14], and because focusing on hairless regions prevented analyses from being influenced by K14-driven overexpression of AREG in the outer root sheath [54]. For all samples, total RNA was extracted using the RNeasy mini kit (Qiagen, Valencia, CA), and after further processing, cDNA was hybridized to Affymetrix 430 2.0 arrays (45,101 probesets).

### Statistical methods

The human and mouse datasets were normalized using the Robust Multichip Average (RMA) algorithm. Following RMA normalization, human data were further processed to calculate expression scores adjusted for gender and batch effects as described by Gudjonsson et al. (51). Analyses were based upon a between-chip mapping of transcripts represented on the Human Genome U133 Plus 2.0 and transcripts on the Affymetrix Mouse Genome 430 2.0 array. This map was downloaded as a single CSV file from the NetAffx analysis center in July 2009 [55], and is based upon reference-sequence similarity from the HomoloGene database [56].

Differential expression of transcripts between human psoriatic skin samples and control skin samples was evaluated using the Limma linear modeling package [57], with P-value adjustment using the Benjamini-Hochberg method [58]. All p-values generated from differential expression analyses were derived from two-tailed hypothesis tests. The over-representation of KEGG and gene ontology terms was evaluated based upon the conditional hypergeometric test procedure implemented in the GOstats package [59], which is available as part of the R Bioconductor software suite [60]. Among transcripts increased or decreased in human psoriasis, a large number of Gene Ontology biological process or KEGG pathway terms were significantly over-represented. Of these, we have focused on that were most frequently associated with the transcripts significantly altered in human psoriasis (i.e., at least 10–12 transcripts per term in Figures 6 and 7; at least 3 transcripts per term in Figures S6 and S7). Correspondence between human-mouse expression patterns with respect to particular Gene Ontology or KEGG pathways was evaluated based upon two-tailed hypothesis tests (i.e., t-tests), which evaluated whether, among transcripts associated with a given term, the mean log_2_-transformed expression difference between psoriasiform and normal mice was greater or less than zero.

## Supporting Information

Figure S1 **Statistically significant correspondence between human psoriasis and mouse psoriasiform phenotypes: Analysis of adjusted residuals.** A total of 33322 human-mouse orthologous transcript pairs were considered, and each pair was assigned to one of nine categories based upon patterns of differential expression. Analyses were repeated with respect to the (A) K5-Tie2 psoriasiform phenotype, (B) IMQ phenotype, (C) K14-AREG phenotype on ear skin, (D) K14-AREG phenotype on tail skin, (E) K5-Stat3C phenotype and (F) K5-TGFβ1 phenotype. In each figure, the nine possible differential expression categories are listed along the horizontal axis, and differ according to whether expression is significantly increased in human psoriasis or the mouse phenotype (▴), significantly decreased in human psoriasis or the mouse phenotype (▾), or not significantly altered in human psoriasis or the mouse phenotype (—). The vertical axis corresponds to the adjusted residual associated with each category. Adjusted residuals quantify the degree to which the observed number of transcript pairs assigned to each category differs from that expected if human and mouse differential expression patterns are unrelated. Positive residuals indicate an overabundance of human-mouse transcript pairs associated with a given category, while negative residuals indicate a lower-than-expected number of human-mouse transcript pairs associated with a given category. Under the null hypothesis, each adjusted residual follows a standard normal distribution, with residuals larger than 3 in absolute value indicative of significant over- or under-abundance. The large positive residuals associated with transcript pairs increased in human psoriasis and mouse psoriasiform phenotypes (6th category from left), as well as decreased in human psoriasis and mouse psoriasiform phenotypes (7th category from left), are therefore suggestive of significant correspondence between expression patterns in human psoriasis and mouse phenotypes. In the case of human psoriasis, transcripts were differentially expressed if the FDR-adjusted p-value was less than 0.05 and the log_2_-transformed fold-change estimate was greater than 1.0 in absolution value. With respect to mouse psoriasiform phenotypes, transcripts were differentially expressed in the comparison-wise p-value was less than 0.05.
(TIF)

Figure S2 **Statistically significant correspondence between human psoriasis and mouse psoriasiform phenotypes: Detection rate curves.** Human transcripts were ranked according to their observed fold change (psoriasis/normal) in descending order, with smaller ranks assigned to transcripts most strongly elevated in psoriasis (sections A, C, E, G, I and K). Alternatively, human transcripts were ranked according to their observed fold change (psoriasis/normal) in ascending order, with smaller ranks assigned to transcripts most strongly decreased in psoriasis (sections B, D, F, H, J and L). For each psoriasiform phenotype, we identified "foreground" gene sets, consisting of the 200 most strongly elevated mouse transcripts (i.e., 200 transcripts with largest psoriasiform/control fold change and P < 0.05; sections A, C, E, G, I and K), or the 200 most strongly decreased mouse transcripts (i.e., 200 transcripts with smallest psoriasiform/control fold change and P < 0.05; sections B, D, F, H, J and L). For each analysis, a "background" set of control transcripts was selected as those mouse transcripts not significantly altered by the psoriasiform phenotype (P>0.20). For both the foreground and background sets of mouse transcripts, each figure shows their cumulate fractional abundance (or detection rate) with respect to the ranked human transcripts. The degree of enrichment corresponds to the area between foreground and background detection curves (i.e., the gray region in each figure). In sections A, C, E, G, I and K, foreground detection curves associated with psoriasiform-increased transcripts are shown in red. In sections B, D, F, H, J and L, foreground detection curves associated with psoriasiform-decreased transcripts are shown in green. In all cases (sections A - L), background detection curves are represented by the black diagonal line.
(TIF)

Figure S3 **Statistically significant correspondence between human psoriasis and mouse psoriasiform phenotypes: Gene set enrichment analysis and enrichment score metric.** A running enrichment score metric was used to evaluate correspondence between gene expression patterns in human psoriasis and the K5-Tie2 phenotype (A and B), IMQ phenotype (C and D), K14-AREG phenotype on ear skin (E and F), K14-AREG phenotype on tail skin (G and H), K5-Stat3C phenotype (I and J) and K5-TGFβ1 phenotype (K and L). Correspondence with respect to psoriasis-increased genes is evaluated in Figures A, C, E, G, I and K, while correspondence with respect to psoriasis-decreased genes is evaluated in Figures B, D, F, H, J and L. Analyses were based upon a total of 15375 human genes and 15602 mouse genes with mouse-human orthology relationships, with expression values for each human and mouse gene summarized by calculating the maximum expression level among multiple transcripts associated with the same gene symbol (see (35)). In each figure, a set *S* was defined, which contained approximately 200 human genes orthologous to the 200 mouse genes most strongly altered by the indicated mouse psoriasiform phenotype (psoriasiform-increased in figures A, C, E, G, I and K; psoriasiform-decreased in figures B, D, F, H, J and L). The horizontal axis corresponds to a ranking of 15375 human genes, with lower ranks assigned to genes most strongly altered in human psoriasis (psoriasis-increased in figures A, C, E, G, I and K; psoriasis-decreased in figures B, D, F, H, J and L). The short vertical lines near the top of the figure mark the rankings of the 200 genes contained in *S* with respect to the 15375 ranked human genes. The vertical axis corresponds to the running enrichment score defined by Subramanian et al. (35). This running score was calculated in cumulative fashion across the 15375 ranked human genes, and increases if a gene is contained in *S*, but decreases if a gene is not contained in *S*. The running score that is greatest in absolute value is defined as the enrichment score (ES) and is circled in red in the figure. The grey region outlines the lower 95% of the null distribution associated with this ES, which was generated using the simulation procedure described by Subramanian et al. (35). Significant enrichment is indicated for cases in which the red circle (ES) lies above the grey region (see p-values in each figure).
(TIF)

Figure S4 **Genes increased in all psoriasiform phenotypes.** We identified a set of 27 mouse genes increased significantly in K5-Tie2, IMQ, K14-AREG (ear), K14-AREG (tail), K5-Stat3C and K5-TGFβ1 psoriasiform phenotypes (P < 0.05 in each comparison, psoriasiform versus control). The identified genes are listed in the left margin of the figure, excluding any mouse genes that could not be mapped to an orthologous human gene. Each point represents the estimated fold change ratio (psoriasiform / control), where different colors correspond to the different mouse phenotypes (see legend). The gray box outlines a 95% confidence interval for the fold change estimate (psoriasis / control) associated with a transcript derived from an orthologous human gene. If a mouse gene was associated with more than one human transcript, the gray box corresponds to the human transcript most significantly altered in clinical psoriasis (i.e., lowest p-value in comparison between psoriasis patients and controls). A simulation analysis indicated that the observed number of mouse genes increased in all models (i.e., 27 genes with P < 0.05 for each of six comparisons and with a human ortholog) was statistically significant (P < 0.001), given the total number of mouse genes significantly increased in each model and the proportion of mouse genes that could be mapped to an ortholgous human gene.
(TIF)

Figure S5 **Genes decreased in all psoriasiform phenotypes.** We identified a set of 44 mouse genes decreased significantly in K5-Tie2, IMQ, K14-AREG (ear), K14-AREG (tail), K5-Stat3C and K5-TGFβ1 psoriasiform phenotypes (P < 0.05 in each comparison, psoriasiform versus control). The identified genes are listed in the left margin of the figure, excluding any mouse genes that could not be mapped to an orthologous human gene. Each point represents the estimated fold change ratio (psoriasiform / control), where different colors correspond to the different mouse phenotypes (see legend). The gray box outlines a 95% confidence interval for the fold change estimate (psoriasis / control) associated with a transcript derived from an orthologous human gene. If a mouse gene was associated with than one human transcript, the gray box corresponds to the human transcript most significantly altered in clinical psoriasis (i.e., lowest p-value in comparison between psoriasis patients and controls). A simulation analysis indicated that the observed number of mouse genes increased in all models (i.e., 44 genes with P < 0.05 for each of six comparisons and with a human ortholog) was statistically significant (P < 0.001), given the total number of mouse genes significantly increased in each model and the proportion of mouse genes that could be mapped to an ortholgous human gene.
(TIF)

Figure S6 **KEGG pathways overrepresented among genes exhibiting increased expression in human psoriasis.** A total of 2617 transcripts were identified as significantly elevated in psoriatic plaques obtained from human patients relative to normal skin obtained from control subjects (FDR-adjusted P < 0.05 and log_2_-transformed fold-change greater than 0.50). These psoriasis-increased transcripts were analyzed to identify KEGG pathway terms significantly over-represented (P < 0.05). For each over-represented term, we determined which of the 2617 transcripts were annotated with the term, and for these human transcripts, we identified a set of mouse transcripts associated with orthologous genes. For each mouse transcript within this set, we calculated the expression ratio between psoriasiform and normal mouse skin, and determined the average value of this ratio among all transcripts in the set. The analysis was repeated with respect to the K5-Tie2 phenotype, IMQ phenotype, K14-AREG phenotype on ear skin, K14-AREG phenotype on tail skin, K5-Stat3C phenotype, and K5-TGFβ1 phenotype. Colors in the chart reflect the average fold-change ratio (psoriasiform / control) of the set of mouse transcripts associated with the KEGG term listed in each row (see scale). Triangle symbols indicate whether the average fold-change estimate associated with mouse transcripts is significantly different from one (see legend; two-sample t-test). The number of transcripts associated with each KEGG term is indicated in brackets (e.g., [*x*/*y*], where *x* denotes the number of human transcripts and *y* represents the number of mouse transcripts derived from orthologous genes). Since mouse transcripts included in this analysis were orthologous to genes exhibiting *elevated* expression in human psoriasis, correspondence between human psoriasis and mouse phenotypes is indicated by red colors and up-triangle symbols.
(TIF)

Figure S7 **KEGG pathways overrepresented among genes exhibiting decreased expression in human psoriasis.** A total of 3540 transcripts were identified as significantly decreased in psoriatic plaques obtained from human patients relative to normal skin obtained from control subjects (FDR-adjusted P < 0.05 and log_2_-transformed fold-change less than -1.0). These psoriasis-decreased transcripts were analyzed to identify KEGG pathways significantly over-represented (P < 0.05). For each over-represented pathway, we determined which of the 3540 transcripts were annotated with the term, and for these human transcripts, we identified a set of mouse transcripts associated with orthologous genes. For each mouse transcript within this set, we calculated the expression ratio between psoriasiform and normal mouse skin, and determined the average value of this ratio among all transcripts in the set. The analysis was repeated with respect to the K5-Tie2 phenotype, IMQ phenotype, K14-AREG phenotype on ear skin, K14-AREG phenotype on tail skin, K5-Stat3C phenotype, and K5-TGFβ1 phenotype. Colors in the chart reflect the average fold-change ratio (psoriasiform / control) of the set of mouse transcripts associated with the gene ontology term listed in each row (see scale). Triangle symbols indicate whether the average fold-change estimate associated with mouse transcripts is significantly different from one (see legend; two-sample t-test). The number of transcripts associated with each KEGG term is indicated in brackets (e.g., [*x*/*y*], where *x* denotes the number of human transcripts and *y* represents the number of mouse transcripts derived from orthologous genes). Since mouse transcripts included in this analysis were orthologous to genes exhibiting *decreased* expression in human psoriasis, correspondence between human psoriasis and mouse phenotypes is indicated by green colors and down-triangle symbols.
(TIF)

Figure S8 **Gene ontology biological processes overrepresented among psoriasis-increased transcripts with correspondent expression patterns among orthologous genes in psoriasiform phenotypes.** We identified 2617 transcripts significantly increased in human psoriasis (FDR-adjusted P < 0.05, log_2_-transformed fold-change estimate greater than 0.50). Among these 2617 transcripts, we isolated those for which expression of mouse orthologues was correspondently elevated in the K5-Tie2 (*n* = 310 transcripts), IMQ (*n*  =  706 transcripts), K14-AREG ear (*n*  =  536 transcripts), K14-AREG tail (*n*  =  909 transcripts), K5-Stat3C (*n*  =  674 transcripts), and K5-TGFβ1 (*n*  =  858 transcripts) psoriasiform phenotypes (comparison-wise P < 0.05). For each set of correspondently altered transcripts, we identified significantly overrepresented gene ontology biological process terms (P < 0.05). In the figure, the identified terms associated with each mouse model have been listed, and terms have been clustered according to the number of ancestors shared between any two gene ontology terms. For each term, the psoriasiform phenotype associated with that term is indicated in parentheses (Tie2  =  K5-Tie2, IMQ  =  imiquimod, AREG-E  =  K14-AREG ear skin, AREG-T  =  K14-AREG tail skin, Stat3C  =  K5-Stat3C, TGF  =  K5-TGFβ1). Terms listed in black font were identified with respect to more than one mouse model and thus represent points of human-mouse correspondence that are shared among psoriasiform phenotypes. Terms listed in colored font correspond to human-mouse similarities that are specific to an individual psoriasiform phenotype.
(TIF)

Figure S9 **Gene ontology biological processes overrepresented among psoriasis-decreased transcripts with correspondent expression patterns among orthologous genes in psoriasiform phenotypes.** We identified 3540 transcripts significantly decreased in human psoriasis (FDR-adjusted P < 0.05, log_2_-transformed fold-change estimate less than -0.50). Among these 3540 transcripts, we isolated those for which expression of mouse orthologues was correspondently decreased in the K5-Tie2 (*n*  =  903 transcripts), IMQ (*n*  =  1126 transcripts), K14-AREG ear (*n*  =  864 transcripts), K14-AREG tail (*n*  =  1127 transcripts), K5-Stat3C (*n*  =  832 transcripts), K5-TGFβ1 (*n*  =  829 transcripts) psoriasiform phenotypes (comparison-wise P < 0.05). For each set of correspondently altered transcripts, we identified significantly overrepresented gene ontology biological process terms (P < 0.05). In the figure, the identified terms associated with each mouse model have been listed, and terms have been clustered according to the number of ancestors shared between any two gene ontology terms. For each term, the psoriasiform phenotype associated with that term is indicated in parentheses (Tie2  =  K5-Tie2, IMQ  =  imiquimod, AREG -E  =  K14-AREG ear skin, AREG -T  =  K14-AREG tail skin, Stat3C  =  K5-Stat3C, TGF  =  K5-TGFβ1). Terms listed in black font were identified with respect to more than one mouse model and thus represent points of human-mouse correspondence that are shared among psoriasiform phenotypes. Terms listed in colored font correspond to human-mouse similarities that are specific to an individual psoriasiform phenotype.
(TIF)

Figure S10 **Gene ontology biological processes overrepresented among psoriasis-increased transcripts with discordant expression patterns among orthologous genes in psoriasiform phenotypes.** We identified 2617 transcripts significantly increased in human psoriasis (FDR-adjusted P < 0.05, log_2_-transformed fold-change estimate greater than 0.50). Among these 2617 transcripts, we isolated those for which expression of mouse orthologues was discordantly decreased in the K5-Tie2 (*n*  =  171 transcripts), IMQ (*n*  =  317 transcripts), K14-AREG ear (*n*  =  165 transcripts), K14-AREG tail (*n*  =  352 transcripts), K5-Stat3C (*n*  =  300 transcripts), K5-TGFβ1 (*n*  =  218 transcripts) psoriasiform phenotypes (comparison-wise P < 0.05). For each set of discordantly altered transcripts, we identified significantly overrepresented gene ontology biological process terms (P < 0.05). In the figure, the identified terms associated with each mouse model have been listed, and terms have been clustered according to the number of ancestors shared between any two gene ontology terms. For each term, the psoriasiform phenotype associated with that term is indicated in parentheses (Tie2  =  K5-Tie2, IMQ  =  imiquimod, AREG -E  =  K14-AREG ear skin, AREG -T  =  K14-AREG tail skin, Stat3C  =  K5-Stat3C, TGF  =  K5-TGFβ1). Terms listed in black font were identified with respect to more than one mouse model and thus represent points of human-mouse discordance that are shared among psoriasiform phenotypes. Terms listed in colored font correspond to human-mouse disparities that are specific to an individual psoriasiform phenotype.
(TIF)

Figure S11 **Gene ontology biological processes overrepresented among psoriasis-decreased transcripts with discordant expression patterns among orthologous genes in psoriasiform phenotypes.** We identified 3540 transcripts significantly decreased in human psoriasis (FDR-adjusted P < 0.05, log_2_-transformed fold-change estimate less than -0.50). Among these 3540 transcripts, we isolated those for which expression of mouse orthologues was discordantly increased in the K5-Tie2 (*n*  =  104 transcripts), IMQ (*n*  =  236 transcripts), K14-AREG ear (*n*  =  170 transcripts), K14-AREG tail (*n*  =  284 transcripts), K5-Stat3C (*n*  =  318 transcripts), K5-TGFβ1 (*n*  =  301 transcripts) psoriasiform phenotypes (comparison-wise P < 0.05). For each set of discordantly altered transcripts, we identified significantly overrepresented GO biological process terms (P < 0.05). In the figure, the identified terms associated with each mouse model have been listed, and terms have been clustered according to the number of ancestors shared between any two gene ontology terms. For each term, the psoriasiform phenotype associated with that term is indicated in parentheses (Tie2  =  K5-Tie2, IMQ  =  imiquimod, AREG -E  =  K14-AREG ear skin, AREG -T  =  K14-AREG tail skin, Stat3C  =  K5-Stat3C, TGF  =  K5-TGFβ1). Terms listed in black font were identified with respect to more than one mouse model and thus represent points of human-mouse discordance that are shared among psoriasiform phenotypes. Terms listed in colored font correspond to human-mouse disparities that are specific to an individual psoriasiform phenotype.
(TIF)

Figure S12 **Mouse orthologs of mitosis-associated genes increased in human psoriasis.** A total of 79 transcripts associated with "mitosis" were significantly elevated in human psoriasis (GO:0007067). With respect to these 79 psoriasis-increased transcripts, we identified 128 corresponding mouse transcripts derived from orthologous mouse genes. A subset of these 128 transcripts is listed in the figure (left margin). The heat map image describes the response patterns of these transcripts in psoriasiform phenotypes relative to normal skin in control mice. Red colors correspond to increased expression in psoriasiform phenotypes and green colors correspond to decreased expression (see scale; right margin). Up- and down-triangles denote transcripts for which the fold-change difference between lesion and normal mouse skin is significant or marginally significant. Since listed transcripts are orthologous to human genes exhibiting increased expression in clinical psoriasis, correspondence to the human disease is denoted by red colors (i.e., increased expression in psoriasiform phenotypes).
(TIF)

Figure S13 **Expression patterns of key cytokines, chemokines, receptors and growth factors in human psoriasis and mouse psoriasiform phenotypes.** The expression patterns of 26 selected genes were examined, where each gene encodes a protein product that is thought to contribute to psoriasis pathogenesis. For a given gene, yellow boxes outline 95% confidence intervals associated with the estimated fold-change (psoriasis / control) in the human disease. Filled circles and standard error bars correspond to fold change estimates (psoriasiform / control) associated with each mouse phenotype (see legend).
(TIF)

## References

[1] Conrad C, Boyman O, Tonel G, Tun-Kyi A, Laggner U (2007). Alpha1beta1 integrin is crucial for accumulation of epidermal T cells and the development of psoriasis.. Nat Med.

[2] Nickoloff BJ, Wrone-Smith T (1999). Injection of pre-psoriatic skin with CD4+ T cells induces psoriasis.. Am J Pathol.

[3] Boehncke WH, Schön MP (2007). Animal models of psoriasis.. Clin Dermatol.

[4] Gudjonsson JE, Johnston A, Dyson M, Valdimarsson H, Elder JT (2007). Mouse models of psoriasis.. J Invest Dermatol.

[5] Nestle FO, Nickoloff BJ (2006). Animal models of psoriasis: a brief update.. J Eur Acad Dermatol Venereol.

[6] Lowe NJ, Breeding J, Kean C, Cohn ML (1981). Psoriasiform dermatosis in a rhesus monkey.. J Invest Dermatol.

[7] Zanolli MD, Jayo MJ, Jayo JM, Blaine D, Hall J (1989). Evaluation of psoriatic plaques that spontaneously developed in a cynomolgus monkey (Macaca fascicularis).. Acta Derm Venereol Suppl (Stockh).

[8] Berking C, Takemoto R, Binder RL, Hartman SM, Ruiter DJ (2002). Photocarcinogenesis in human adult skin grafts.. Carcinogenesis.

[9] Khavari PA (2006). Modelling cancer in human skin tissue.. Nat Rev Cancer.

[10] Godfrey DI, Hammond KJ, Poulton LD, Smyth MJ, Baxter AG (2000). NKT cells: facts, functions and fallacies.. Immunol Today.

[11] Ardavín C (2003). Origin, precursors and differentiation of mouse dendritic cells.. Nat Rev Immunol.

[12] Wolfram JA, Diaconu D, Hatala DA, Rastegar J, Knutsen DA (2009). Keratinocyte but not endothelial cell-specific overexpression of Tie2 leads to the development of psoriasis.. Am J Pathol.

[13] van der Fits L, Mourits S, Voerman JS, Kant M, Boon L (2009). Imiquimod-induced psoriasis-like skin inflammation in mice is mediated via the IL-23/IL-17 axis.. J Immunol.

[14] Cook PW, Piepkorn M, Clegg CH, Plowman GD, DeMay JM (1997). Transgenic expression of the human amphiregulin gene induces a psoriasis-like phenotype.. J Clin Invest.

[15] Sano S, Chan KS, Carbajal S, Clifford J, Peavey M (2005). Stat3 links activated keratinocytes and immunocytes required for development of psoriasis in a novel transgenic mouse model.. Nat Med.

[16] Li AG, Wang D, Feng XH, Wang XJ (2004). Latent TGFbeta1 overexpression in keratinocytes results in a severe psoriasis-like skin disorder.. EMBO J.

[17] Gilliet M, Conrad C, Geiges M, Cozzio A, Thürlimann W (2004). Psoriasis triggered by toll-like receptor 7 agonist imiquimod in the presence of dermal plasmacytoid dendritic cell precursors.. Arch Dermatol.

[18] Wu JK, Siller G, Strutton G (2004). Psoriasis induced by topical imiquimod.. Australas J Dermatol.

[19] Rajan N, Langtry JA (2006). Generalized exacerbation of psoriasis associated with imiquimod cream treatment of superficial basal cell carcinomas.. Clin Exp Dermatol.

[20] Fanti PA, Dika E, Vaccari S, Miscial C, Varotti C (2006). Generalized psoriasis induced by topical treatment of actinic keratosis with imiquimod.. Int J Dermatol.

[21] Bowcock AM, Shannon W, Du F, Duncan J, Cao K (2001). Insights into psoriasis and other inflammatory diseases from large-scale gene expression studies.. Hum Mol Genet.

[22] Zhou X, Krueger JG, Kao MC, Lee E, Du F (2003). Novel mechanisms of T-cell and dendritic cell activation revealed by profiling of psoriasis on the 63,100-element oligonucleotide array.. Physiol Genomics.

[23] Kulski JK, Kenworthy W, Bellgard M, Taplin R, Okamoto K (2005). Gene expression profiling of Japanese psoriatic skin reveals an increased activity in molecular stress and immune response signals.. J Mol Med.

[24] Romanowska M, al Yacoub N, Seidel H, Donandt S, Gerken H (2008). PPARdelta enhances keratinocyte proliferation in psoriasis and induces heparin-binding EGF-like growth factor.. J Invest Dermatol.

[25] Nair RP, Duffin KC, Helms C, Ding J, Stuart PE (2009). Genome-wide scan reveals association of psoriasis with IL-23 and NF-kappaB pathways.. Nat Genet.

[26] Strange A, Capon F, Spencer CC, Knight J, Genetic Analysis of Psoriasis Consortium & the Wellcome Trust Case Control Consortium 2 (2010). A genome-wide association study identifies new psoriasis susceptibility loci and an interaction between HLA-C and ERAP1.. Nat Genet.

[27] Stuart PE, Nair RP, Ellinghaus E, Ding J, Tejasvi T (2010). Genome-wide association analysis identifies three psoriasis susceptibility loci.. Nat Genet.

[28] Sun LD, Cheng H, Wang ZX, Zhang AP, Wang PG (2010). Association analyses identify six new psoriasis susceptibility loci in the Chinese population.. Nat Genet.

[29] Hüffmeier U, Uebe S, Ekici AB, Bowes J, Giardina E (2010). Common variants at TRAF3IP2 are associated with susceptibility to psoriatic arthritis and psoriasis.. Nat Genet.

[30] Zhang XJ, Huang W, Yang S, Sun LD, Zhang FY (2009). Psoriasis genome-wide association study identifies susceptibility variants within LCE gene cluster at 1q21.. Nat Genet.

[31] Swindell WR, Johnston A, Gudjonsson JE (2010). Transcriptional profiles of leukocyte populations provide a tool for interpreting gene expression patterns associated with high fat diet in mice.. PLoS One.

[32] Lottaz C, Yang X, Scheid S, Spang R (2006). OrderedList—a bioconductor package for detecting similarity in ordered gene lists.. Bioinformatics.

[33] Swindell WR (2009). Genes and gene expression modules associated with caloric restriction and aging in the laboratory mouse.. BMC Genomics.

[34] Philippakis AA, Busser BW, Gisselbrecht SS, He FS, Estrada B (2006). Expression-guided in silico evaluation of candidate cis regulatory codes for Drosophila muscle founder cells.. PLoS Comput Biol.

[35] Subramanian A, Tamayo P, Mootha VK, Mukherjee S, Ebert BL (2005). Gene set enrichment analysis: a knowledge-based approach for interpreting genome-wide expression profiles.. Proc Natl Acad Sci USA.

[36] Zenaro E, Donini M, Dusi S (2009). Induction of Th1/Th17 immune response by Mycobacterium tuberculosis: role of dectin-1, Mannose Receptor, and DC-SIGN.. J Leukoc Biol.

[37] Haider AS, Lowes MA, Suàrez-Fariñas M, Zaba LC, Cardinale I (2008). Cellular genomic maps help dissect pathology in human skin disease.. J Invest Dermatol.

[38] Nestle FO, Kaplan DH, Barker J (2009). Psoriasis.. N Engl J Med.

[39] Gürtl B, Kratky D, Guelly C, Zhang L, Gorkiewicz G (2009). Apoptosis and fibrosis are early features of heart failure in an animal model of metabolic cardiomyopathy.. Int J Exp Pathol.

[40] Janus C, Westaway D (2001). Transgenic mouse models of Alzheimer's disease.. Physiol Behav.

[41] Allen TJ, Cooper ME, Lan HY (2004). Use of genetic mouse models in the study of diabetic nephropathy.. Curr Diab Rep.

[42] Kim S (2009). Animal models of cancer in the head and neck region.. Clin Exp Otorhinolaryngol.

[43] Seredkina N, Zykova SN, Rekvig OP (2009). Progression of murine lupus nephritis is linked to acquired renal Dnase1 deficiency and not to up-regulated apoptosis.. Am J Pathol.

[44] Asquith DL, Miller AM, McInnes IB, Liew FY (2009). Animal models of rheumatoid arthritis.. Eur J Immunol.

[45] Elder JT, Bruce AT, Gudjonsson JE, Johnston A, Stuart PE (2010). Molecular dissection of psoriasis: integrating genetics and biology.. J Invest Dermatol.

[46] Van Joost T, Bos JD, Heule F, Meinardi MM (1988). Low-dose cyclosporin A in severe psoriasis. A double-blind study.. Br J Dermatol.

[47] Eedy DJ, Burrows D, Bridges JM, Jones FG (1990). Clearance of severe psoriasis after allogenic bone marrow transplantation.. BMJ.

[48] Ward NL, Loyd CM, Wolfram JA, Diaconu D, Michaels CM (2010). Depletion of antigen presenting cells by clodronate liposomes reverses the psoriatic skin phenotype in KC-Tie2 mice.. Br J Dermatol.

[49] Kircik LH, Del Rosso JQ (2009). Anti-TNF agents for the treatment of psoriasis.. J Drugs Dermatol.

[50] Haider AS, Cohen J, Fei J, Zaba LC, Cardinale I (2008). Insights into gene modulation by therapeutic TNF and IFNgamma antibodies: TNF regulates IFNgamma production by T cells and TNF-regulated genes linked to psoriasis transcriptome.. J Invest Dermatol.

[51] Gudjonsson JE, Ding J, Li X, Nair RP, Tejasvi T (2009). Global Gene Expression Analysis Reveals Evidence for Decreased Lipid Biosynthesis and Increased Innate Immunity in Uninvolved Psoriatic Skin.. J Invest Dermatol.

[52] Diamond I, Owolabi T, Marco M, Lam C, Glick A (2000). Conditional gene expression in the epidermis of transgenic mice using the tetracycline-regulated transactivators tTA and rTA linked to the keratin 5 promoter.. J Invest Dermatol.

[53] Jones N, Voskas D, Master Z, Sarao R, Jones J (2001). Rescue of the early vascular defects in Tek/Tie2 null mice reveals an essential survival function.. EMBO Rep.

[54] Coulombe PA, Kopan R, Fuchs E (1989). Expression of keratin K14 in the epidermis and hair follicle: insights into complex programs of differentiation.. J Cell Biol.

[55] Liu G, Loraine AE, Shigeta R, Cline M, Cheng J (2003). NetAffx: Affymetrix probesets and annotations.. Nucleic Acids Res.

[56] Sayers EW, Barrett T, Benson DA, Bryant SH, Canese K (2009). Database resources of the National Center for Biotechnology Information.. Nucleic Acids Res.

[57] Smyth GK (2004). Linear models and empirical bayes methods for assessing differential expression in microarray experiments.. Stat Appl Genet Mol Biol.

[58] Benjamini Y, Hochberg Y (1995). Controlling the false discovery rate: a powerful and practical approach to multiple testing.. J Roy Stat Soc B.

[59] Falcon S, Gentleman R (2007). Using GOstats to test gene lists for GO term association.. Bioinformatics.

[60] Reimers M, Carey VJ (2006). Bioconductor: an open source framework for bioinformatics and computational biology.. Methods Enzymol.

