# Supplementary figures and images for: Genome-Wide Expression Profiling of Five Mouse Models Identifies Similarities and Differences with Human Psoriasis
Source: PLoS One. 2011 Apr 4;6(4):e18266. doi: 10.1371/journal.pone.0018266 (PMC3070727; doi:10.1371/journal.pone.0018266)

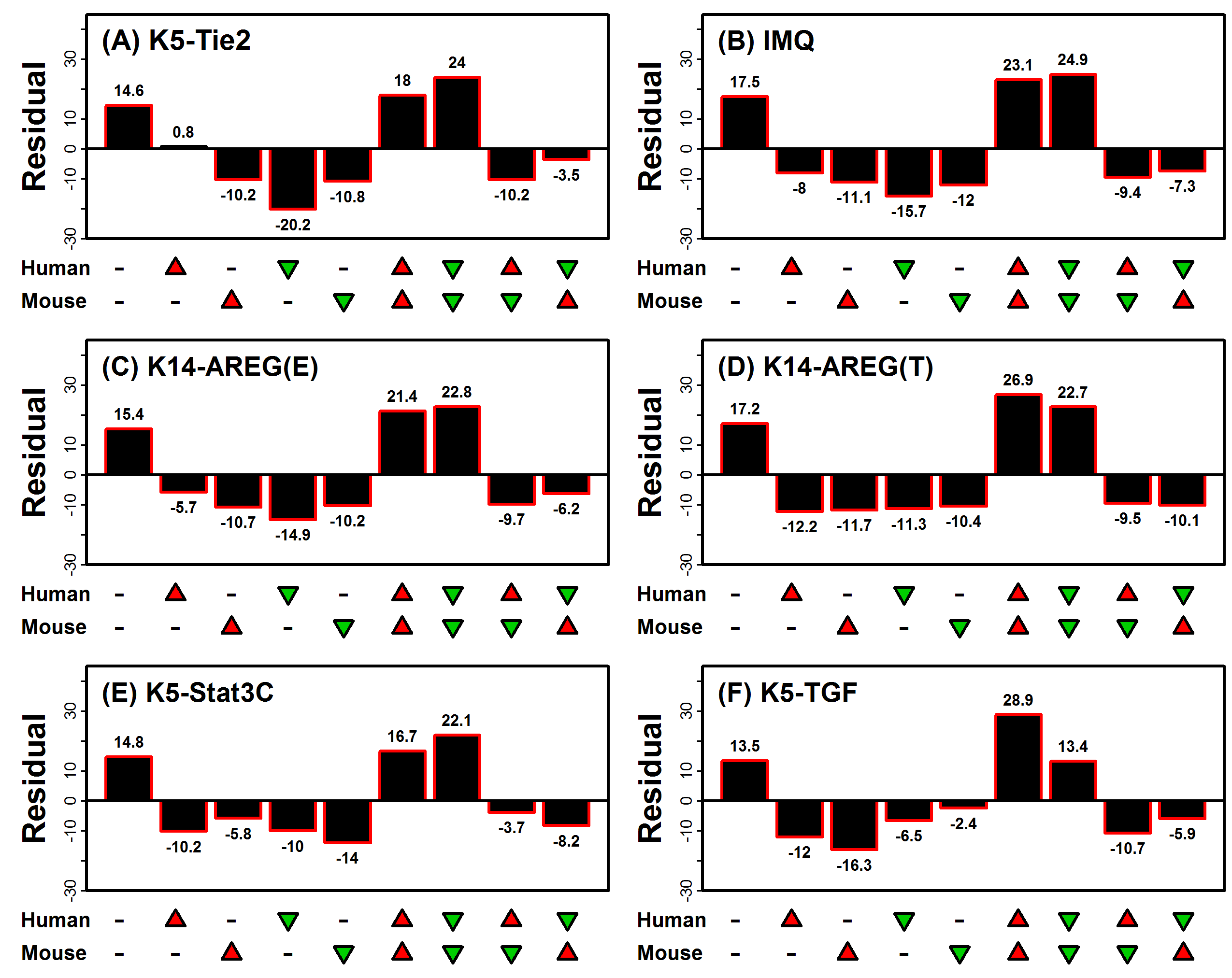

Supplement: Figure S1 — Statistically significant correspondence between human psoriasis and mouse psoriasiform phenotypes: Analysis of adjusted residuals. A total of 33322 human-mouse orthologous transcript pairs were considered, and each pair was assigned to one of nine categories based upon patterns of differential expression. Analyses were repeated with respect to the (A) K5-Tie2 psoriasiform phenotype, (B) IMQ phenotype, (C) K14-AREG phenotype on ear skin, (D) K14-AREG phenotype on tail skin, (E) K5-Stat3C phenotype and (F) K5-TGFβ1 phenotype. In each figure, the nine possible differential expression categories are listed along the horizontal axis, and differ according to whether expression is significantly increased in human psoriasis or the mouse phenotype (▴), significantly decreased in human psoriasis or the mouse phenotype (▾), or not significantly altered in human psoriasis or the mouse phenotype (—). The vertical axis corresponds to the adjusted residual associated with each category. Adjusted residuals quantify the degree to which the observed number of transcript pairs assigned to each category differs from that expected if human and mouse differential expression patterns are unrelated. Positive residuals indicate an overabundance of human-mouse transcript pairs associated with a given category, while negative residuals indicate a lower-than-expected number of human-mouse transcript pairs associated with a given category. Under the null hypothesis, each adjusted residual follows a standard normal distribution, with residuals larger than 3 in absolute value indicative of significant over- or under-abundance. The large positive residuals associated with transcript pairs increased in human psoriasis and mouse psoriasiform phenotypes (6th category from left), as well as decreased in human psoriasis and mouse psoriasiform phenotypes (7th category from left), are therefore suggestive of significant correspondence between expression patterns in human psoriasis and mouse phenotypes. [file pone.0018266.s001.tif]

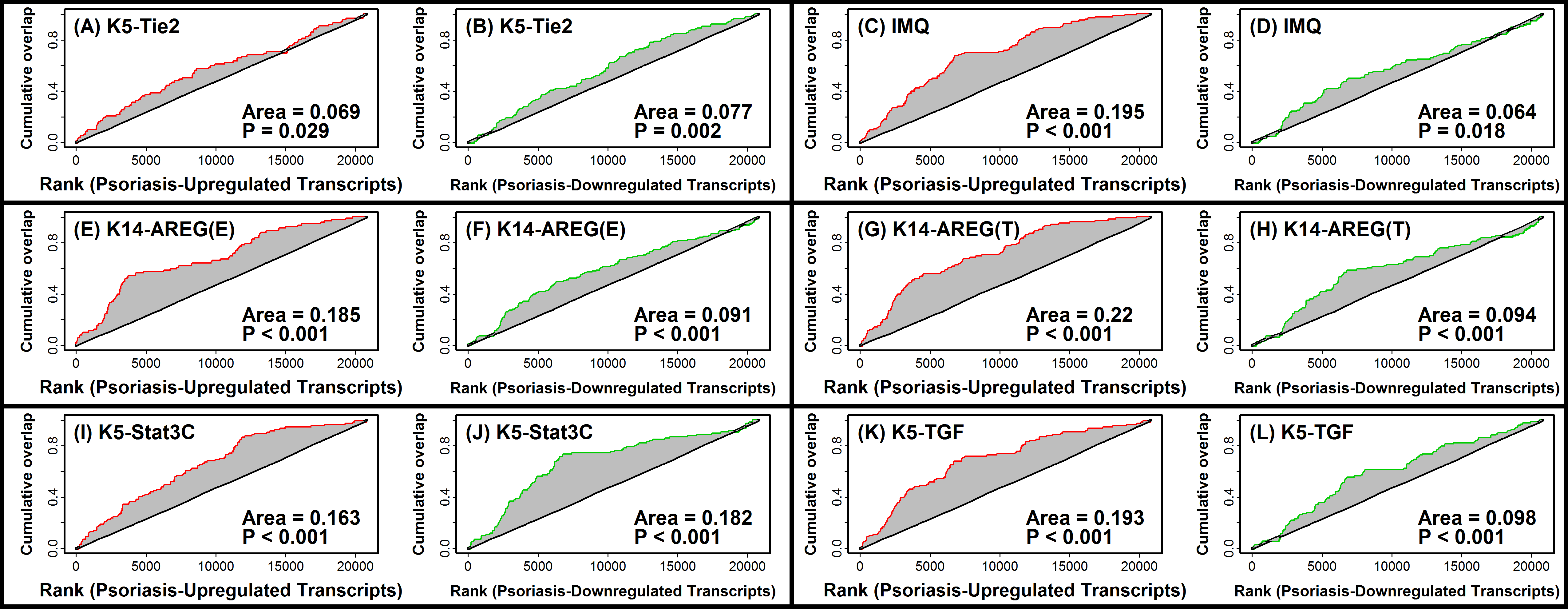

Supplement: Figure S2 — Statistically significant correspondence between human psoriasis and mouse psoriasiform phenotypes: Detection rate curves. Human transcripts were ranked according to their observed fold change (psoriasis/normal) in descending order, with smaller ranks assigned to transcripts most strongly elevated in psoriasis (sections A, C, E, G, I and K). Alternatively, human transcripts were ranked according to their observed fold change (psoriasis/normal) in ascending order, with smaller ranks assigned to transcripts most strongly decreased in psoriasis (sections B, D, F, H, J and L). For each psoriasiform phenotype, we identified "foreground" gene sets, consisting of the 200 most strongly elevated mouse transcripts (i.e., 200 transcripts with largest psoriasiform/control fold change and P < 0.05; sections A, C, E, G, I and K), or the 200 most strongly decreased mouse transcripts (i.e., 200 transcripts with smallest psoriasiform/control fold change and P < 0.05; sections B, D, F, H, J and L). For each analysis, a "background" set of control transcripts was selected as those mouse transcripts not significantly altered by the psoriasiform phenotype (P>0.20). For both the foreground and background sets of mouse transcripts, each figure shows their cumulate fractional abundance (or detection rate) with respect to the ranked human transcripts. The degree of enrichment corresponds to the area between foreground and background detection curves (i.e., the gray region in each figure). In sections A, C, E, G, I and K, foreground detection curves associated with psoriasiform-increased transcripts are shown in red. In sections B, D, F, H, J and L, foreground detection curves associated with psoriasiform-decreased transcripts are shown in green. In all cases (sections A - L), background detection curves are represented by the black diagonal line. (TIF) [file pone.0018266.s002.tif]

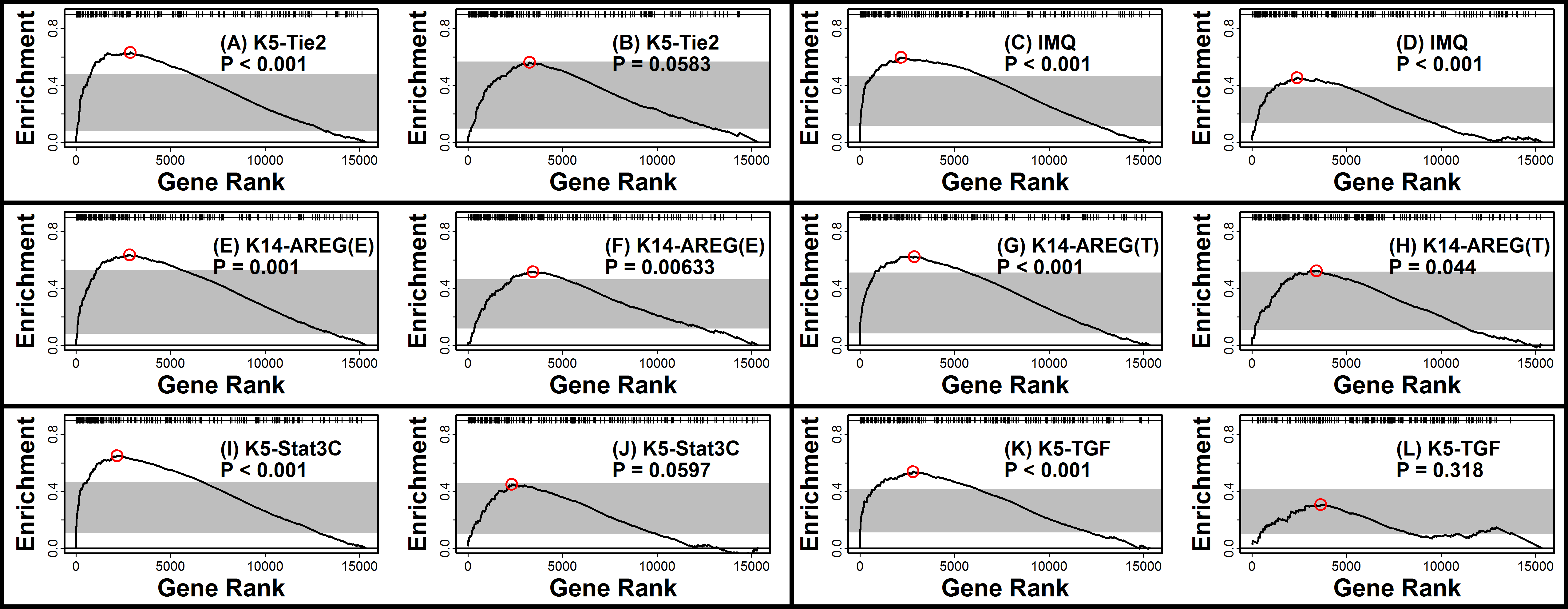

Supplement: Figure S3 — Statistically significant correspondence between human psoriasis and mouse psoriasiform phenotypes: Gene set enrichment analysis and enrichment score metric. A running enrichment score metric was used to evaluate correspondence between gene expression patterns in human psoriasis and the K5-Tie2 phenotype (A and B), IMQ phenotype (C and D), K14-AREG phenotype on ear skin (E and F), K14-AREG phenotype on tail skin (G and H), K5-Stat3C phenotype (I and J) and K5-TGFβ1 phenotype (K and L). Correspondence with respect to psoriasis-increased genes is evaluated in Figures A, C, E, G, I and K, while correspondence with respect to psoriasis-decreased genes is evaluated in Figures B, D, F, H, J and L. Analyses were based upon a total of 15375 human genes and 15602 mouse genes with mouse-human orthology relationships, with expression values for each human and mouse gene summarized by calculating the maximum expression level among multiple transcripts associated with the same gene symbol (see (35)). In each figure, a set S was defined, which contained approximately 200 human genes orthologous to the 200 mouse genes most strongly altered by the indicated mouse psoriasiform phenotype (psoriasiform-increased in figures A, C, E, G, I and K; psoriasiform-decreased in figures B, D, F, H, J and L). The horizontal axis corresponds to a ranking of 15375 human genes, with lower ranks assigned to genes most strongly altered in human psoriasis (psoriasis-increased in figures A, C, E, G, I and K; psoriasis-decreased in figures B, D, F, H, J and L). The short vertical lines near the top of the figure mark the rankings of the 200 genes contained in S with respect to the 15375 ranked human genes. The vertical axis corresponds to the running enrichment score defined by Subramanian et al. (35). This running score was calculated in cumulative fashion across the 15375 ranked human genes, and increases if a gene is contained in S, but decreases if a gene is not contained in S. The running score tha [file pone.0018266.s003.tif]

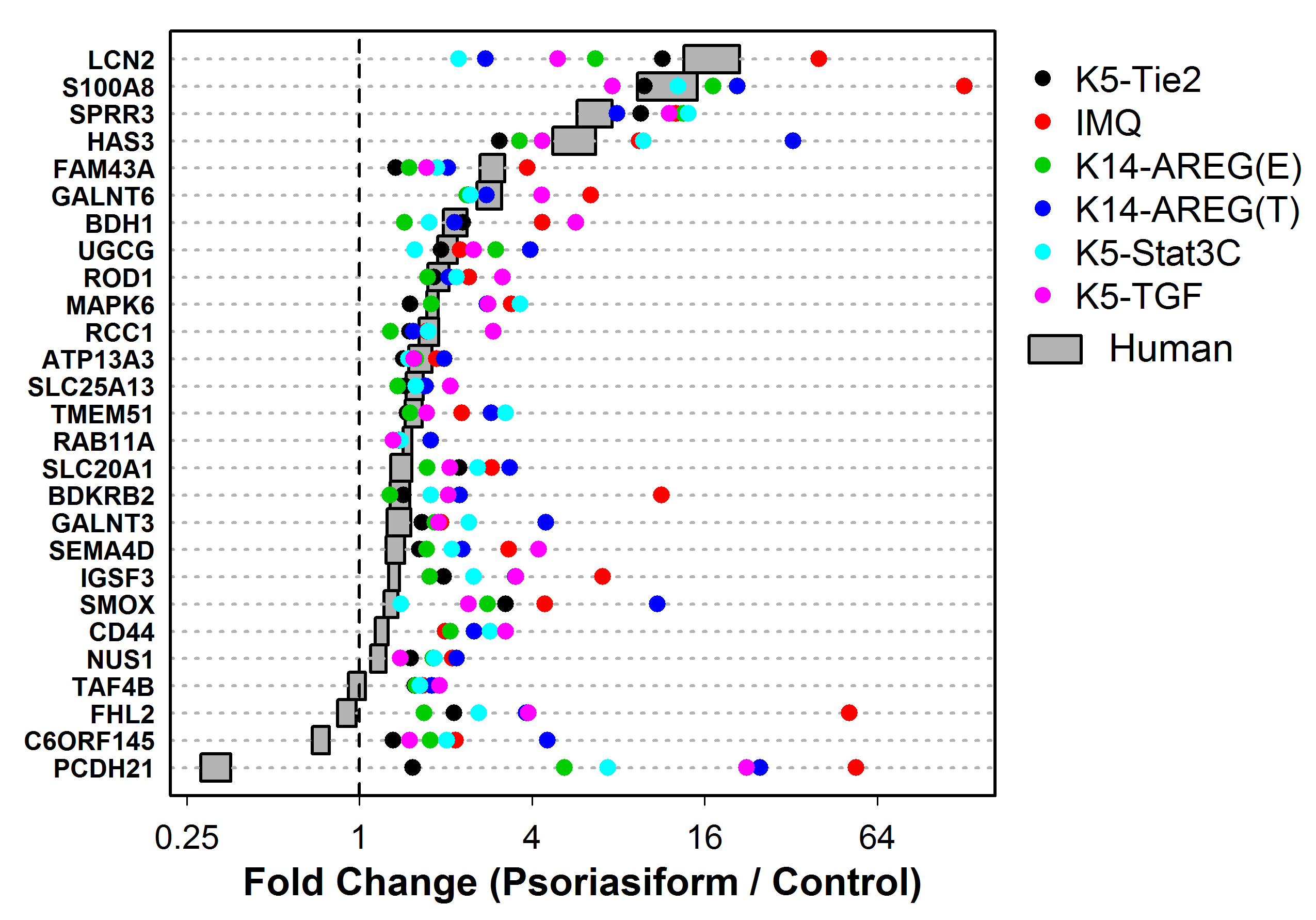

Supplement: Figure S4 — Genes increased in all psoriasiform phenotypes. We identified a set of 27 mouse genes increased significantly in K5-Tie2, IMQ, K14-AREG (ear), K14-AREG (tail), K5-Stat3C and K5-TGFβ1 psoriasiform phenotypes (P < 0.05 in each comparison, psoriasiform versus control). The identified genes are listed in the left margin of the figure, excluding any mouse genes that could not be mapped to an orthologous human gene. Each point represents the estimated fold change ratio (psoriasiform / control), where different colors correspond to the different mouse phenotypes (see legend). The gray box outlines a 95% confidence interval for the fold change estimate (psoriasis / control) associated with a transcript derived from an orthologous human gene. If a mouse gene was associated with more than one human transcript, the gray box corresponds to the human transcript most significantly altered in clinical psoriasis (i.e., lowest p-value in comparison between psoriasis patients and controls). A simulation analysis indicated that the observed number of mouse genes increased in all models (i.e., 27 genes with P < 0.05 for each of six comparisons and with a human ortholog) was statistically significant (P < 0.001), given the total number of mouse genes significantly increased in each model and the proportion of mouse genes that could be mapped to an ortholgous human gene. (TIF) [file pone.0018266.s004.tif]

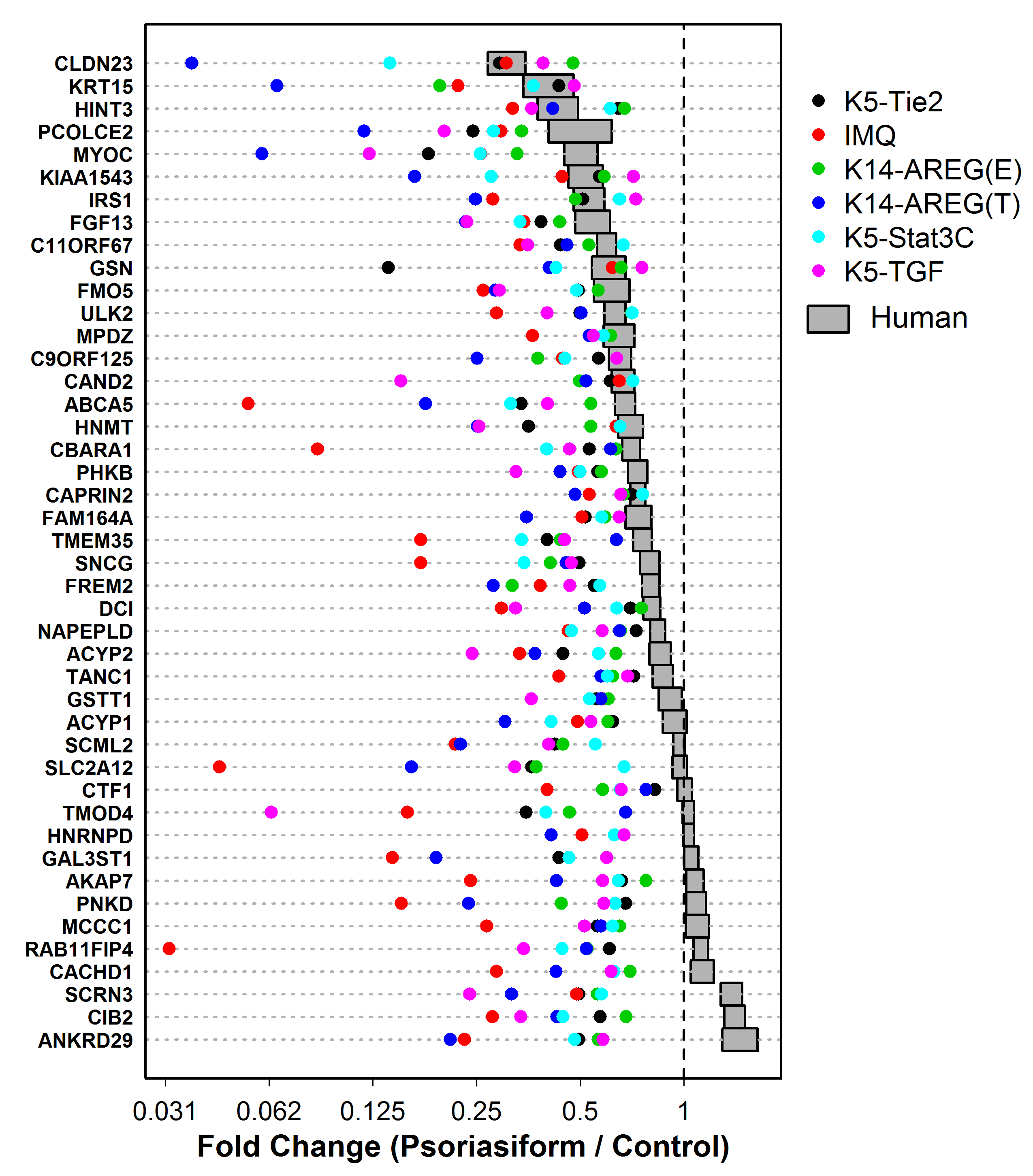

Supplement: Figure S5 — Genes decreased in all psoriasiform phenotypes. We identified a set of 44 mouse genes decreased significantly in K5-Tie2, IMQ, K14-AREG (ear), K14-AREG (tail), K5-Stat3C and K5-TGFβ1 psoriasiform phenotypes (P < 0.05 in each comparison, psoriasiform versus control). The identified genes are listed in the left margin of the figure, excluding any mouse genes that could not be mapped to an orthologous human gene. Each point represents the estimated fold change ratio (psoriasiform / control), where different colors correspond to the different mouse phenotypes (see legend). The gray box outlines a 95% confidence interval for the fold change estimate (psoriasis / control) associated with a transcript derived from an orthologous human gene. If a mouse gene was associated with than one human transcript, the gray box corresponds to the human transcript most significantly altered in clinical psoriasis (i.e., lowest p-value in comparison between psoriasis patients and controls). A simulation analysis indicated that the observed number of mouse genes increased in all models (i.e., 44 genes with P < 0.05 for each of six comparisons and with a human ortholog) was statistically significant (P < 0.001), given the total number of mouse genes significantly increased in each model and the proportion of mouse genes that could be mapped to an ortholgous human gene. (TIF) [file pone.0018266.s005.tif]

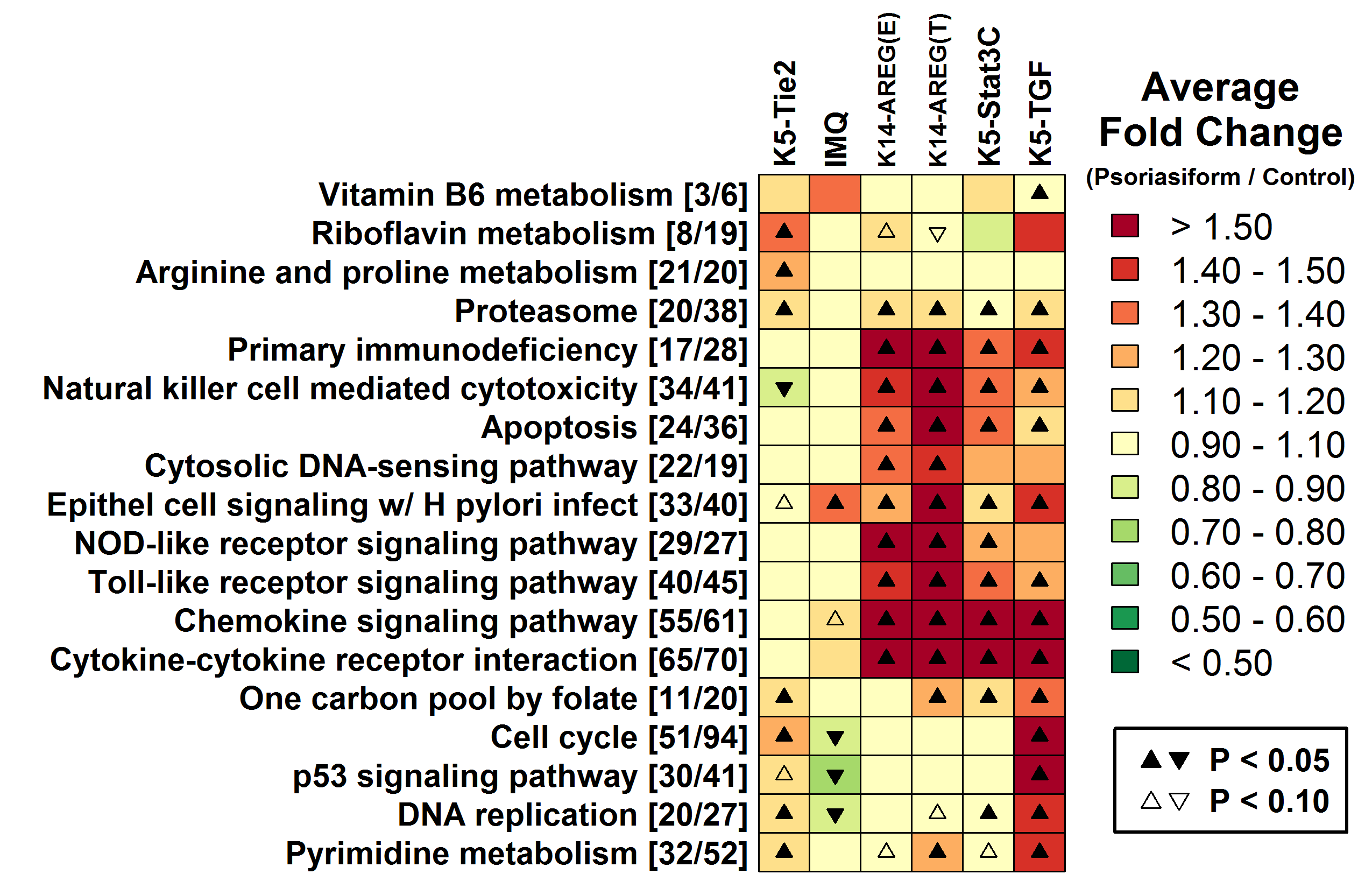

Supplement: Figure S6 — KEGG pathways overrepresented among genes exhibiting increased expression in human psoriasis. A total of 2617 transcripts were identified as significantly elevated in psoriatic plaques obtained from human patients relative to normal skin obtained from control subjects (FDR-adjusted P < 0.05 and log2-transformed fold-change greater than 0.50). These psoriasis-increased transcripts were analyzed to identify KEGG pathway terms significantly over-represented (P < 0.05). For each over-represented term, we determined which of the 2617 transcripts were annotated with the term, and for these human transcripts, we identified a set of mouse transcripts associated with orthologous genes. For each mouse transcript within this set, we calculated the expression ratio between psoriasiform and normal mouse skin, and determined the average value of this ratio among all transcripts in the set. The analysis was repeated with respect to the K5-Tie2 phenotype, IMQ phenotype, K14-AREG phenotype on ear skin, K14-AREG phenotype on tail skin, K5-Stat3C phenotype, and K5-TGFβ1 phenotype. Colors in the chart reflect the average fold-change ratio (psoriasiform / control) of the set of mouse transcripts associated with the KEGG term listed in each row (see scale). Triangle symbols indicate whether the average fold-change estimate associated with mouse transcripts is significantly different from one (see legend; two-sample t-test). The number of transcripts associated with each KEGG term is indicated in brackets (e.g., [x/y], where x denotes the number of human transcripts and y represents the number of mouse transcripts derived from orthologous genes). Since mouse transcripts included in this analysis were orthologous to genes exhibiting elevated expression in human psoriasis, correspondence between human psoriasis and mouse phenotypes is indicated by red colors and up-triangle symbols. (TIF) [file pone.0018266.s006.tif]

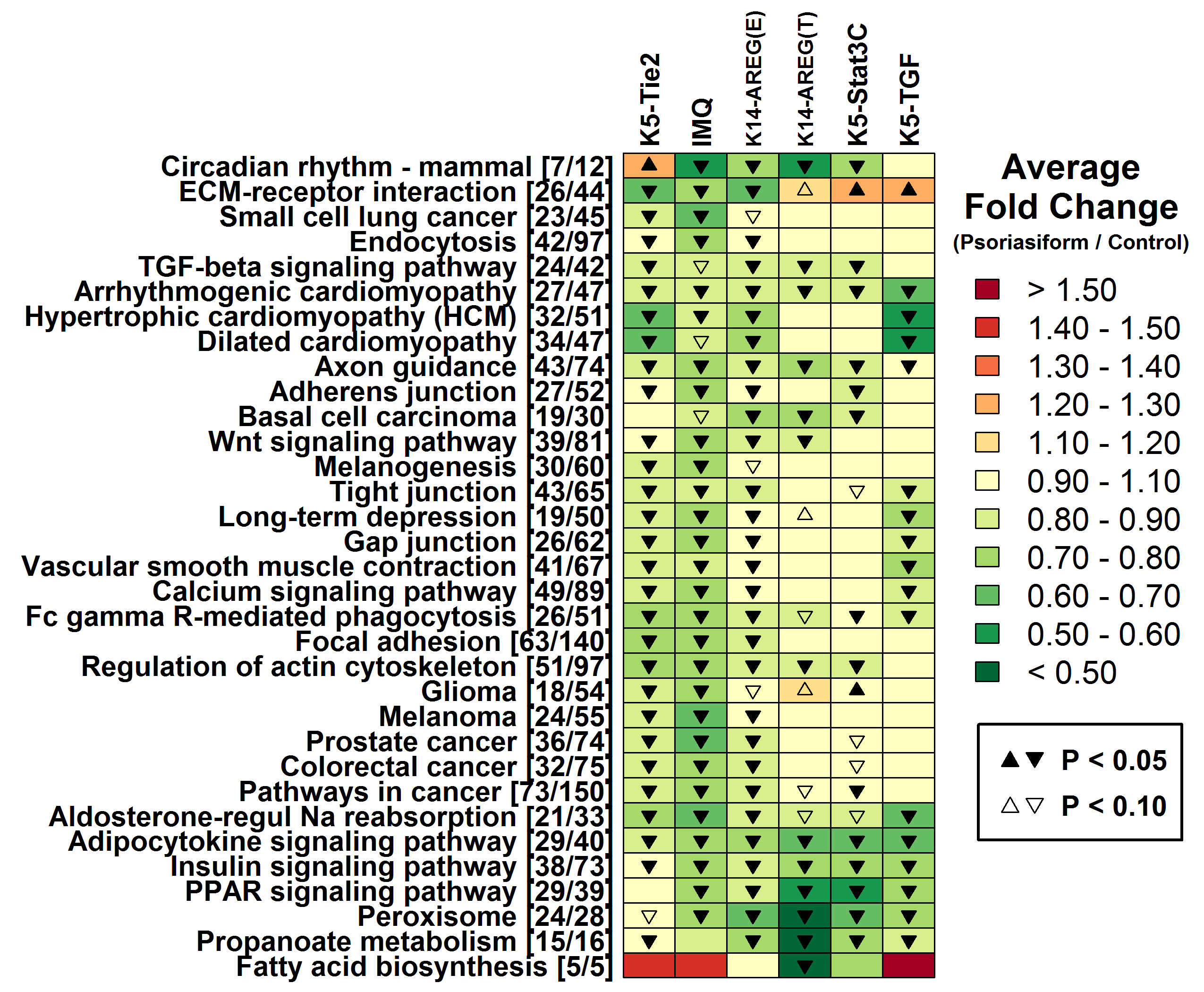

Supplement: Figure S7 — KEGG pathways overrepresented among genes exhibiting decreased expression in human psoriasis. A total of 3540 transcripts were identified as significantly decreased in psoriatic plaques obtained from human patients relative to normal skin obtained from control subjects (FDR-adjusted P < 0.05 and log2-transformed fold-change less than -1.0). These psoriasis-decreased transcripts were analyzed to identify KEGG pathways significantly over-represented (P < 0.05). For each over-represented pathway, we determined which of the 3540 transcripts were annotated with the term, and for these human transcripts, we identified a set of mouse transcripts associated with orthologous genes. For each mouse transcript within this set, we calculated the expression ratio between psoriasiform and normal mouse skin, and determined the average value of this ratio among all transcripts in the set. The analysis was repeated with respect to the K5-Tie2 phenotype, IMQ phenotype, K14-AREG phenotype on ear skin, K14-AREG phenotype on tail skin, K5-Stat3C phenotype, and K5-TGFβ1 phenotype. Colors in the chart reflect the average fold-change ratio (psoriasiform / control) of the set of mouse transcripts associated with the gene ontology term listed in each row (see scale). Triangle symbols indicate whether the average fold-change estimate associated with mouse transcripts is significantly different from one (see legend; two-sample t-test). The number of transcripts associated with each KEGG term is indicated in brackets (e.g., [x/y], where x denotes the number of human transcripts and y represents the number of mouse transcripts derived from orthologous genes). Since mouse transcripts included in this analysis were orthologous to genes exhibiting decreased expression in human psoriasis, correspondence between human psoriasis and mouse phenotypes is indicated by green colors and down-triangle symbols. (TIF) [file pone.0018266.s007.tif]

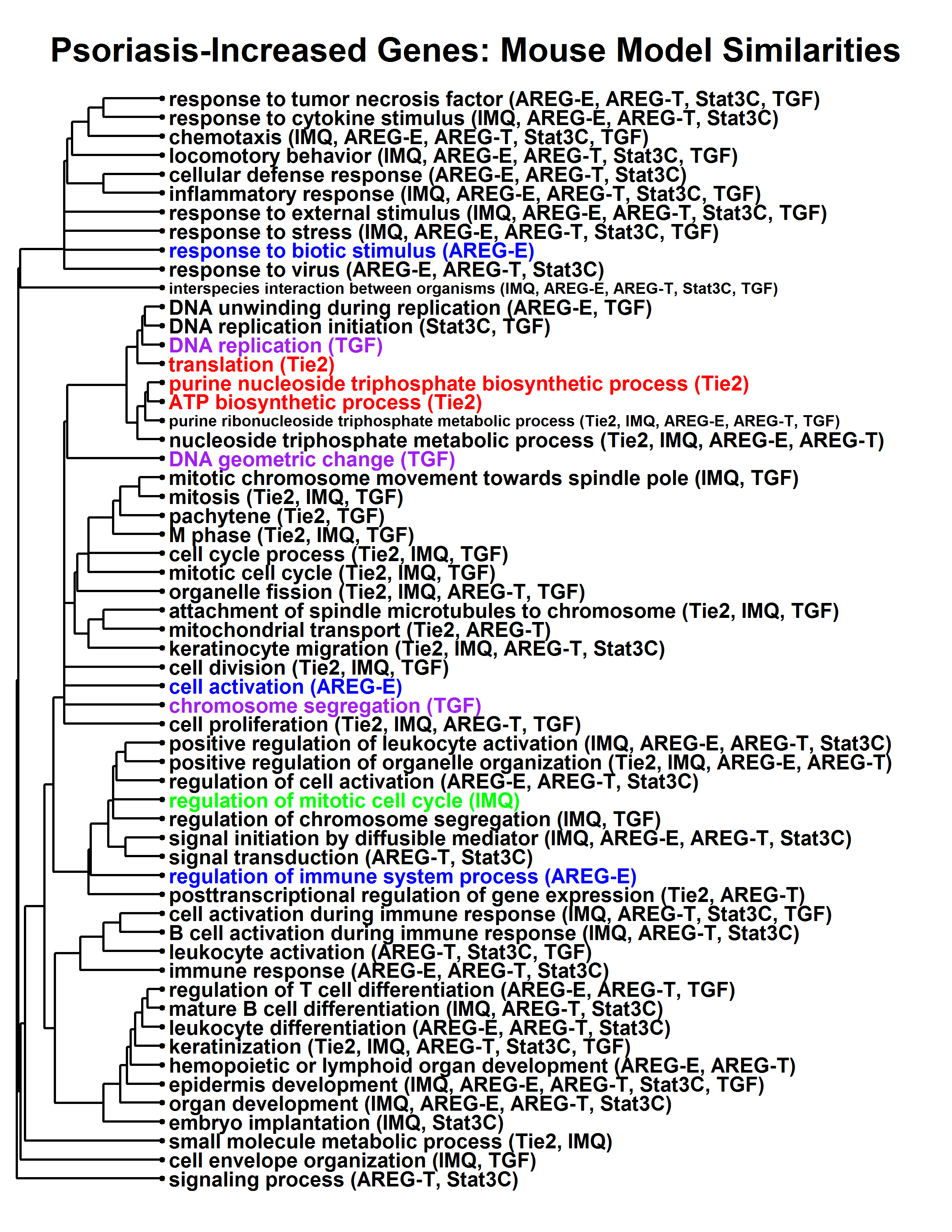

Supplement: Figure S8 — Gene ontology biological processes overrepresented among psoriasis-increased transcripts with correspondent expression patterns among orthologous genes in psoriasiform phenotypes. We identified 2617 transcripts significantly increased in human psoriasis (FDR-adjusted P < 0.05, log2-transformed fold-change estimate greater than 0.50). Among these 2617 transcripts, we isolated those for which expression of mouse orthologues was correspondently elevated in the K5-Tie2 (n = 310 transcripts), IMQ (n = 706 transcripts), K14-AREG ear (n = 536 transcripts), K14-AREG tail (n = 909 transcripts), K5-Stat3C (n = 674 transcripts), and K5-TGFβ1 (n = 858 transcripts) psoriasiform phenotypes (comparison-wise P < 0.05). For each set of correspondently altered transcripts, we identified significantly overrepresented gene ontology biological process terms (P < 0.05). In the figure, the identified terms associated with each mouse model have been listed, and terms have been clustered according to the number of ancestors shared between any two gene ontology terms. For each term, the psoriasiform phenotype associated with that term is indicated in parentheses (Tie2 = K5-Tie2, IMQ = imiquimod, AREG-E = K14-AREG ear skin, AREG-T = K14-AREG tail skin, Stat3C = K5-Stat3C, TGF = K5-TGFβ1). Terms listed in black font were identified with respect to more than one mouse model and thus represent points of human-mouse correspondence that are shared among psoriasiform phenotypes. Terms listed in colored font correspond to human-mouse similarities that are specific to an individual psoriasiform phenotype. (TIF) [file pone.0018266.s008.tif]

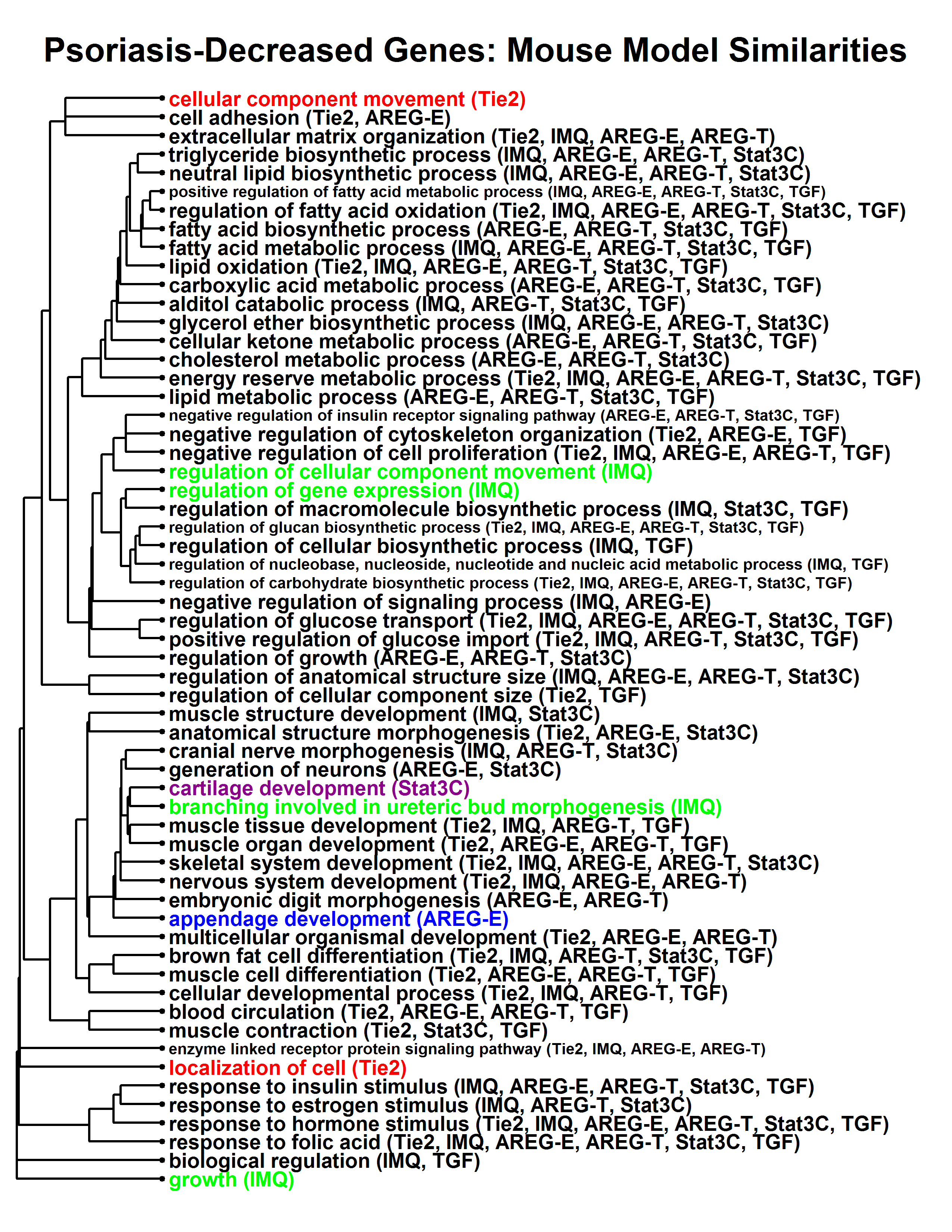

Supplement: Figure S9 — Gene ontology biological processes overrepresented among psoriasis-decreased transcripts with correspondent expression patterns among orthologous genes in psoriasiform phenotypes. We identified 3540 transcripts significantly decreased in human psoriasis (FDR-adjusted P < 0.05, log2-transformed fold-change estimate less than -0.50). Among these 3540 transcripts, we isolated those for which expression of mouse orthologues was correspondently decreased in the K5-Tie2 (n = 903 transcripts), IMQ (n = 1126 transcripts), K14-AREG ear (n = 864 transcripts), K14-AREG tail (n = 1127 transcripts), K5-Stat3C (n = 832 transcripts), K5-TGFβ1 (n = 829 transcripts) psoriasiform phenotypes (comparison-wise P < 0.05). For each set of correspondently altered transcripts, we identified significantly overrepresented gene ontology biological process terms (P < 0.05). In the figure, the identified terms associated with each mouse model have been listed, and terms have been clustered according to the number of ancestors shared between any two gene ontology terms. For each term, the psoriasiform phenotype associated with that term is indicated in parentheses (Tie2 = K5-Tie2, IMQ = imiquimod, AREG -E = K14-AREG ear skin, AREG -T = K14-AREG tail skin, Stat3C = K5-Stat3C, TGF = K5-TGFβ1). Terms listed in black font were identified with respect to more than one mouse model and thus represent points of human-mouse correspondence that are shared among psoriasiform phenotypes. Terms listed in colored font correspond to human-mouse similarities that are specific to an individual psoriasiform phenotype. (TIF) [file pone.0018266.s009.tif]

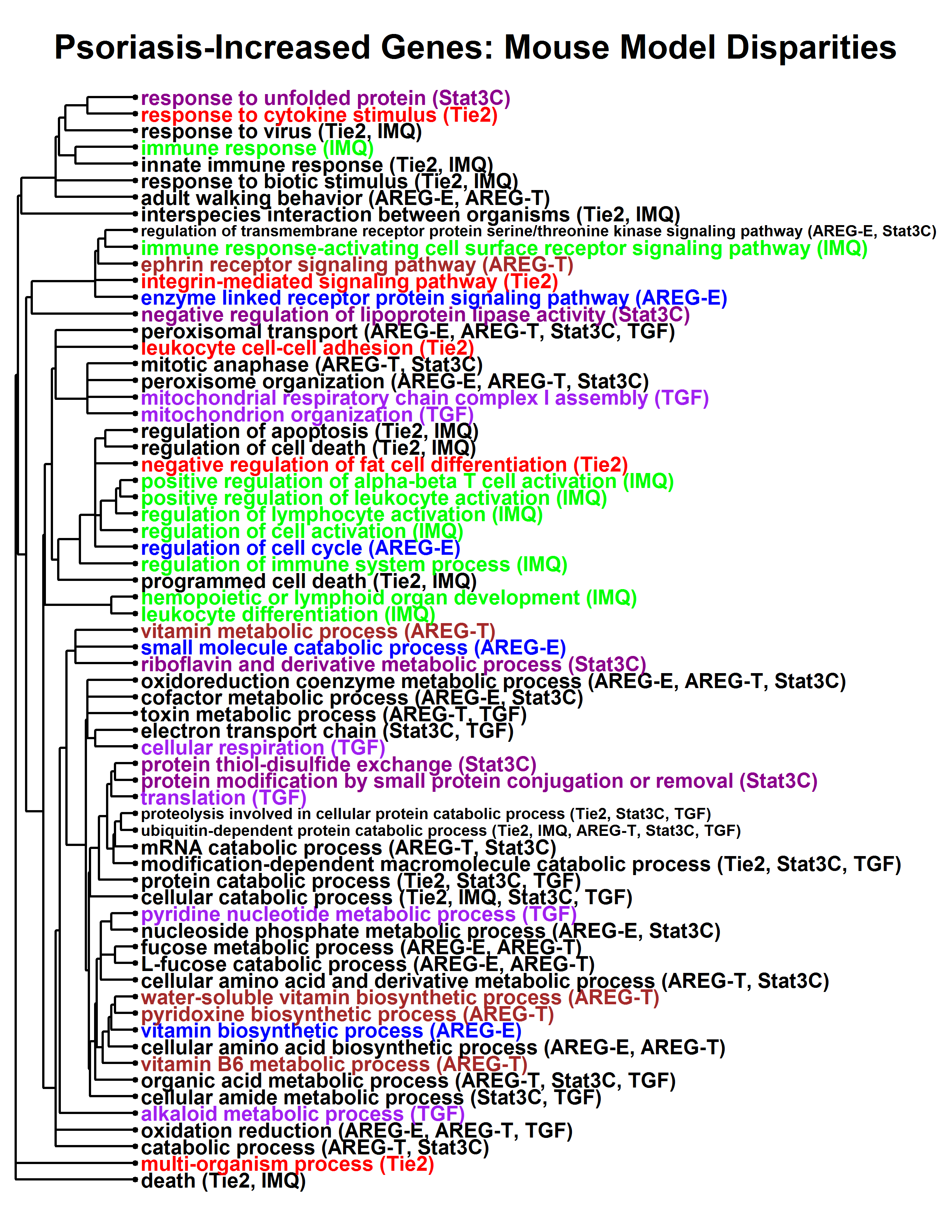

Supplement: Figure S10 — Gene ontology biological processes overrepresented among psoriasis-increased transcripts with discordant expression patterns among orthologous genes in psoriasiform phenotypes. We identified 2617 transcripts significantly increased in human psoriasis (FDR-adjusted P < 0.05, log2-transformed fold-change estimate greater than 0.50). Among these 2617 transcripts, we isolated those for which expression of mouse orthologues was discordantly decreased in the K5-Tie2 (n = 171 transcripts), IMQ (n = 317 transcripts), K14-AREG ear (n = 165 transcripts), K14-AREG tail (n = 352 transcripts), K5-Stat3C (n = 300 transcripts), K5-TGFβ1 (n = 218 transcripts) psoriasiform phenotypes (comparison-wise P < 0.05). For each set of discordantly altered transcripts, we identified significantly overrepresented gene ontology biological process terms (P < 0.05). In the figure, the identified terms associated with each mouse model have been listed, and terms have been clustered according to the number of ancestors shared between any two gene ontology terms. For each term, the psoriasiform phenotype associated with that term is indicated in parentheses (Tie2 = K5-Tie2, IMQ = imiquimod, AREG -E = K14-AREG ear skin, AREG -T = K14-AREG tail skin, Stat3C = K5-Stat3C, TGF = K5-TGFβ1). Terms listed in black font were identified with respect to more than one mouse model and thus represent points of human-mouse discordance that are shared among psoriasiform phenotypes. Terms listed in colored font correspond to human-mouse disparities that are specific to an individual psoriasiform phenotype. (TIF) [file pone.0018266.s010.tif]

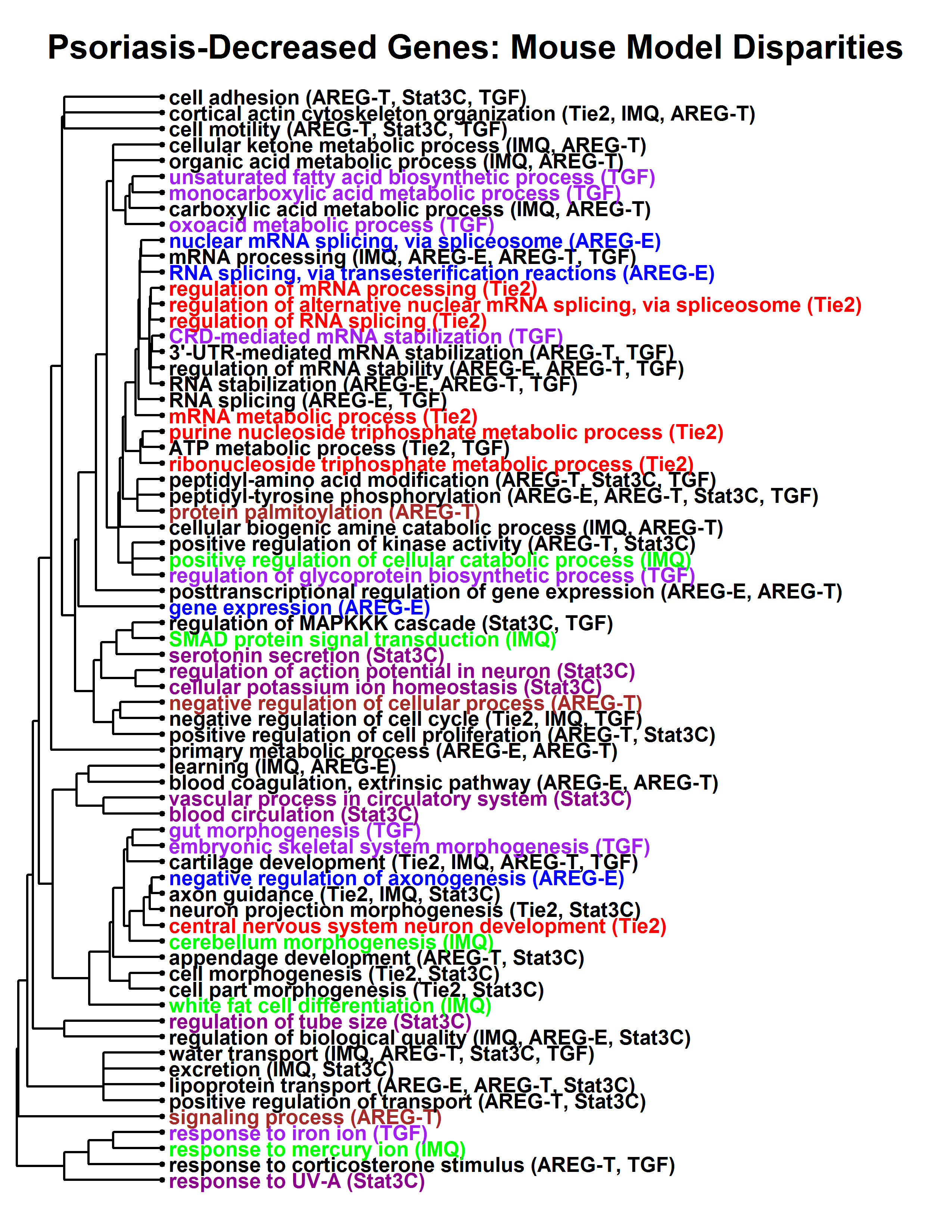

Supplement: Figure S11 — Gene ontology biological processes overrepresented among psoriasis-decreased transcripts with discordant expression patterns among orthologous genes in psoriasiform phenotypes. We identified 3540 transcripts significantly decreased in human psoriasis (FDR-adjusted P < 0.05, log2-transformed fold-change estimate less than -0.50). Among these 3540 transcripts, we isolated those for which expression of mouse orthologues was discordantly increased in the K5-Tie2 (n = 104 transcripts), IMQ (n = 236 transcripts), K14-AREG ear (n = 170 transcripts), K14-AREG tail (n = 284 transcripts), K5-Stat3C (n = 318 transcripts), K5-TGFβ1 (n = 301 transcripts) psoriasiform phenotypes (comparison-wise P < 0.05). For each set of discordantly altered transcripts, we identified significantly overrepresented GO biological process terms (P < 0.05). In the figure, the identified terms associated with each mouse model have been listed, and terms have been clustered according to the number of ancestors shared between any two gene ontology terms. For each term, the psoriasiform phenotype associated with that term is indicated in parentheses (Tie2 = K5-Tie2, IMQ = imiquimod, AREG -E = K14-AREG ear skin, AREG -T = K14-AREG tail skin, Stat3C = K5-Stat3C, TGF = K5-TGFβ1). Terms listed in black font were identified with respect to more than one mouse model and thus represent points of human-mouse discordance that are shared among psoriasiform phenotypes. Terms listed in colored font correspond to human-mouse disparities that are specific to an individual psoriasiform phenotype. (TIF) [file pone.0018266.s011.tif]

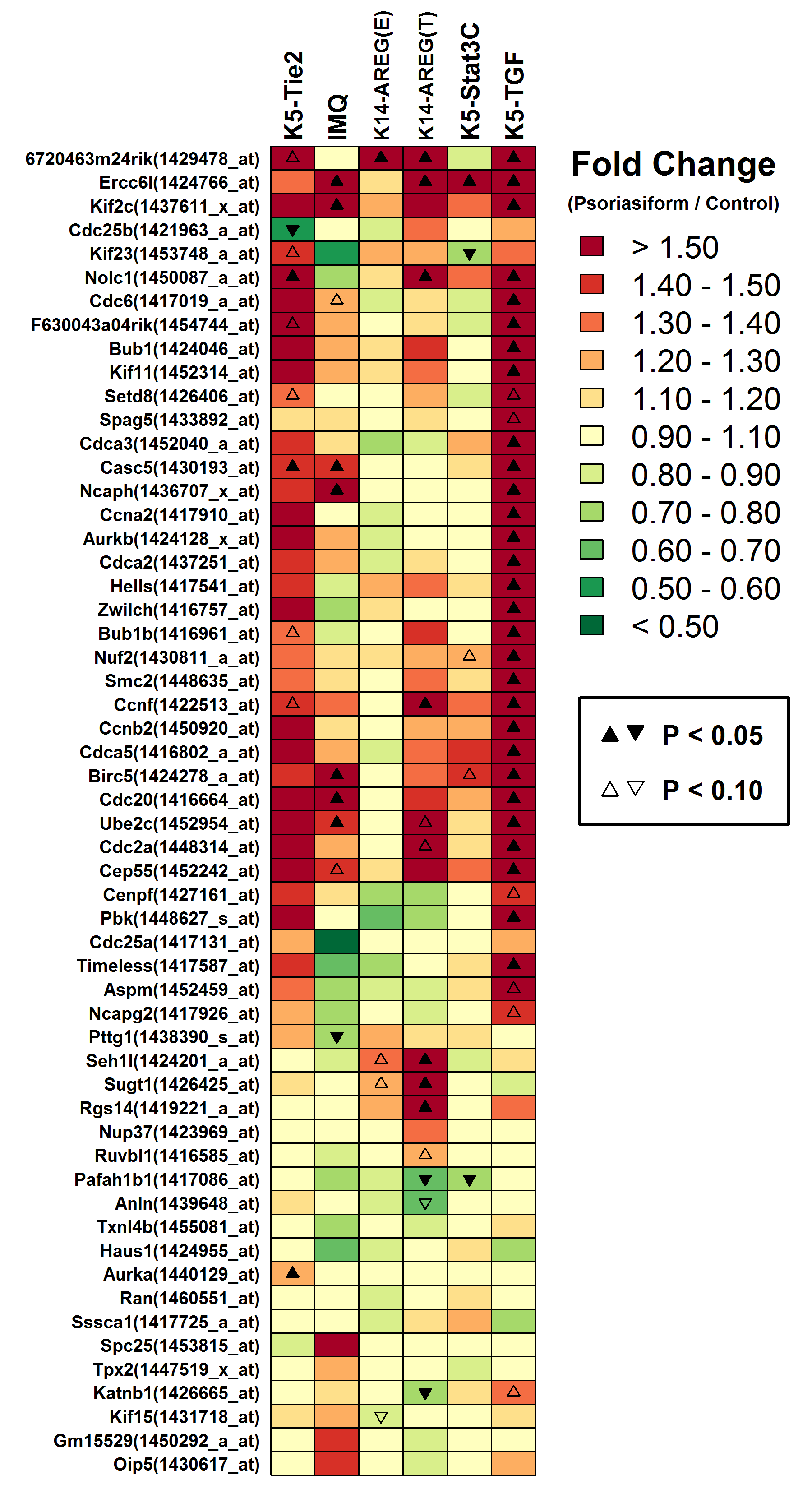

Supplement: Figure S12 — Mouse orthologs of mitosis-associated genes increased in human psoriasis. A total of 79 transcripts associated with "mitosis" were significantly elevated in human psoriasis (GO:0007067). With respect to these 79 psoriasis-increased transcripts, we identified 128 corresponding mouse transcripts derived from orthologous mouse genes. A subset of these 128 transcripts is listed in the figure (left margin). The heat map image describes the response patterns of these transcripts in psoriasiform phenotypes relative to normal skin in control mice. Red colors correspond to increased expression in psoriasiform phenotypes and green colors correspond to decreased expression (see scale; right margin). Up- and down-triangles denote transcripts for which the fold-change difference between lesion and normal mouse skin is significant or marginally significant. Since listed transcripts are orthologous to human genes exhibiting increased expression in clinical psoriasis, correspondence to the human disease is denoted by red colors (i.e., increased expression in psoriasiform phenotypes). (TIF) [file pone.0018266.s012.tif]

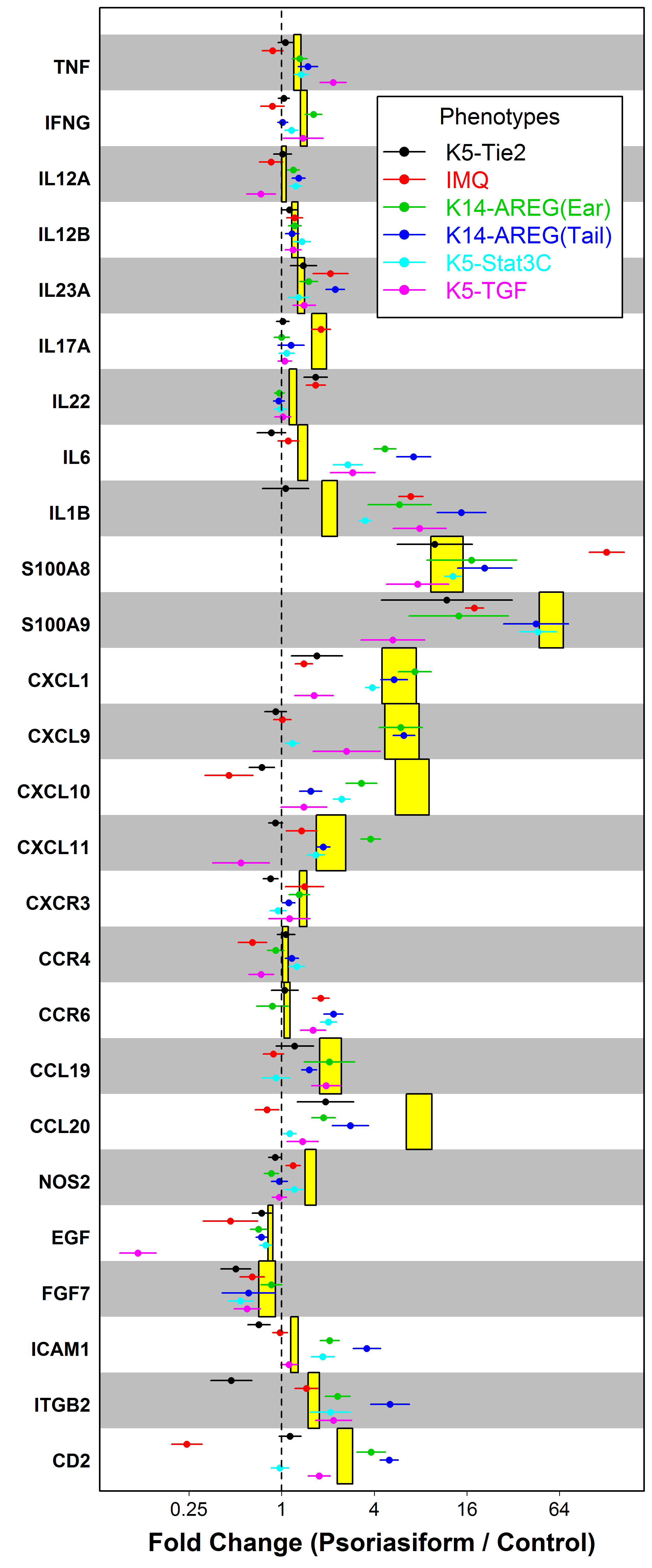

Supplement: Figure S13 — Expression patterns of key cytokines, chemokines, receptors and growth factors in human psoriasis and mouse psoriasiform phenotypes. The expression patterns of 26 selected genes were examined, where each gene encodes a protein product that is thought to contribute to psoriasis pathogenesis. For a given gene, yellow boxes outline 95% confidence intervals associated with the estimated fold-change (psoriasis / control) in the human disease. Filled circles and standard error bars correspond to fold change estimates (psoriasiform / control) associated with each mouse phenotype (see legend). (TIF) [file pone.0018266.s013.tif]
